# Supplementary material for: Neurotrophic and Immunomodulatory Lanostane Triterpenoids from Wood-Inhabiting Basidiomycota
Source: Int J Mol Sci. 2022 Nov 6;23(21):13593. doi: 10.3390/ijms232113593 (PMC9657622; doi:10.3390/ijms232113593)
Supplement: Supplementary file 1 [file ijms-23-13593-s001.zip › ijms-1913099-supplementary.pdf]

Supplementary materials

Neurotrophic and Immunomodulatory Lanostane Triterpenoids from Wood-Inhabiting Basidiomycota

Khadija Hassan[1,2], Blondelle Matio Kemkuignou[1,2], Marco Kirchenwitz[3] , Kathrin Wittstein[1,2], Monique Rascher-Albaghdadi[1,4] , Clara Chepkirui[1,2], Josphat C. Matasyoh[5], Cony Decock[6], Reinhard W. Köster[4], Theresia E. B. Stradal[3], Marc Stadler[1,2]*

[1] Department of Microbial Drugs, Helmholtz Centre for Infection Research (HZI), German Centre for Infection Research (DZIF), Partner Site Hannover/Braunschweig, Inhoffenstrasse 7, 38124 Braunschweig, Germany

2 Institute of Microbiology, Technische Universität Braunschweig, Spielmannstraße 7, 38106 Braun-schweig, Germany

3 Department of Cell Biology, Helmholtz Centre for Infection Research, Inhoffenstrasse 7, 38124 Braun-schweig, Germany

4 Department of Cellular and Molecular Neurobiology, Zoological Institute, Technische Universität Braunschweig, Spielmannstraße 7, 38106 Braunschweig, Germany

5 Department of Chemistry, Egerton University, P.O. Box 536, 20115, Njoro, Kenya

6 Mycothéque de l'Université Catholique de Louvain (BCCM/MUCL), Place Croix du Sud 3, B-1348 Louvain-la-Neuve, Belgium

*** Correspondence: Marc.Stadler@helmholtz-hzi.de; Tel.: +49-531-6181-424**

Table of Contents

Figure S1: ESIMS data for tumulosic acid (**8**). 3

Figure S2: HR-ESIMS data for tumulosic acid (**8**). 4

Figure S3: ^{1}H NMR spectrum (DMSO-d_6, 700 MHz) of tumulosic acid (**8**). 5

Figure S4: COSY spectrum (DMSO-d6, 700 MHz) of tumulosic acid (**8**). 6

Figure S5: HSQC spectrum (DMSO-d6, 700 MHz) of tumulosic acid (**8**). 7

Figure S6: HMBC spectrum (DMSO-d_6, 700 MHz) of tumulosic acid (**8**). 8

Figure S7: ESIMS data for polyporenic acid C (**9**). 9

Figure S8: HR-ESIMS data for polyporenic acid C (**9**). 10

Figure S9: ^{1}H NMR spectrum (DMSO-d_6, 700 MHz) of polyporenic acid C (**9**). 11

Figure S10: COSY spectrum (DMSO-d_6, 700 MHz) of polyporenic acid C (**9**). 12

Figure S11: HSQC spectrum (DMSO-d_6, 700 MHz) of polyporenic acid C (**9**). 13

Figure S12: HMBC spectrum (DMSO-d_6, 700 MHz) of polyporenic acid C (**9**). 14

Figure S13: ESIMS data for 16α-hydroxyeburiconic acid (**10**). 15

Figure S14: HR-ESIMS data for 16α-hydroxyeburiconic acid (**10**). 16

Figure S15: ^{1}H NMR spectrum (DMSO-d_6, 700 MHz) of 16α-hydroxyeburiconic acid (**10**). 17

Figure S16: COSY spectrum (DMSO-d6, 700 MHz) of 16α-hydroxyeburiconic acid (**10**). 18

Figure S17: HSQC spectrum (DMSO-d_6, 700 MHz) of 16α-hydroxyeburiconic acid (**10**). 19

Figure S18: HMBC spectrum (DMSO-d_6, 700 MHz) of 16α-hydroxyeburiconic acid (**10**). 20

Figure S19: ROESY spectrum (DMSO-d_6, 700 MHz) of 16α-hydroxyeburiconic acid (**10**). 21

Figure S20: ESIMS data for dehydrotumulosic acid (**11**) 22

Figure S21: HR-ESIMS data for dehydrotumulosic acid (**11**). 23

Figure S22: ^{1}H NMR spectrum (DMSO-d_6, 700 MHz) of dehydrotumulosic acid (**11**). 24

Figure S23: COSY spectrum (DMSO-d_6, 700 MHz) of dehydrotumulosic acid (**11**). 25

Figure S24: HSQC spectrum (DMSO-d_6, 700 MHz) of dehydrotumulosic acid (**11**). 26

Figure S25: HMBC spectrum (DMSO-d_6, 700 MHz) of dehydrotumulosic acid (**11**). 27

Figure S26: ESIMS data for pachymic acid (**12**) 28

Figure S27: HR-ESIMS data for pachymic acid (**12**) 29

Figure 28: 1H NMR spectrum (DMSO-d6, 700 MHz) of pachymic acid (**12**). 30

Figure S29: COSY spectrum (DMSO-d_6, 700 MHz) of pachymic acid (**12**). 31

Figure S30: HSQC spectrum (DMSO-d_6, 700 MHz) of pachymic acid (**12**). 32

Figure S31: HMBC spectrum (DMSO-d_6, 700 MHz) of pachymic acid (**12**). 33

Figure S32: ROESY spectrum (DMSO-d_6, 700 MHz) of pachymic acid (**12**). 34

ITS and LSU sequences of *Antrodia* sp. 35

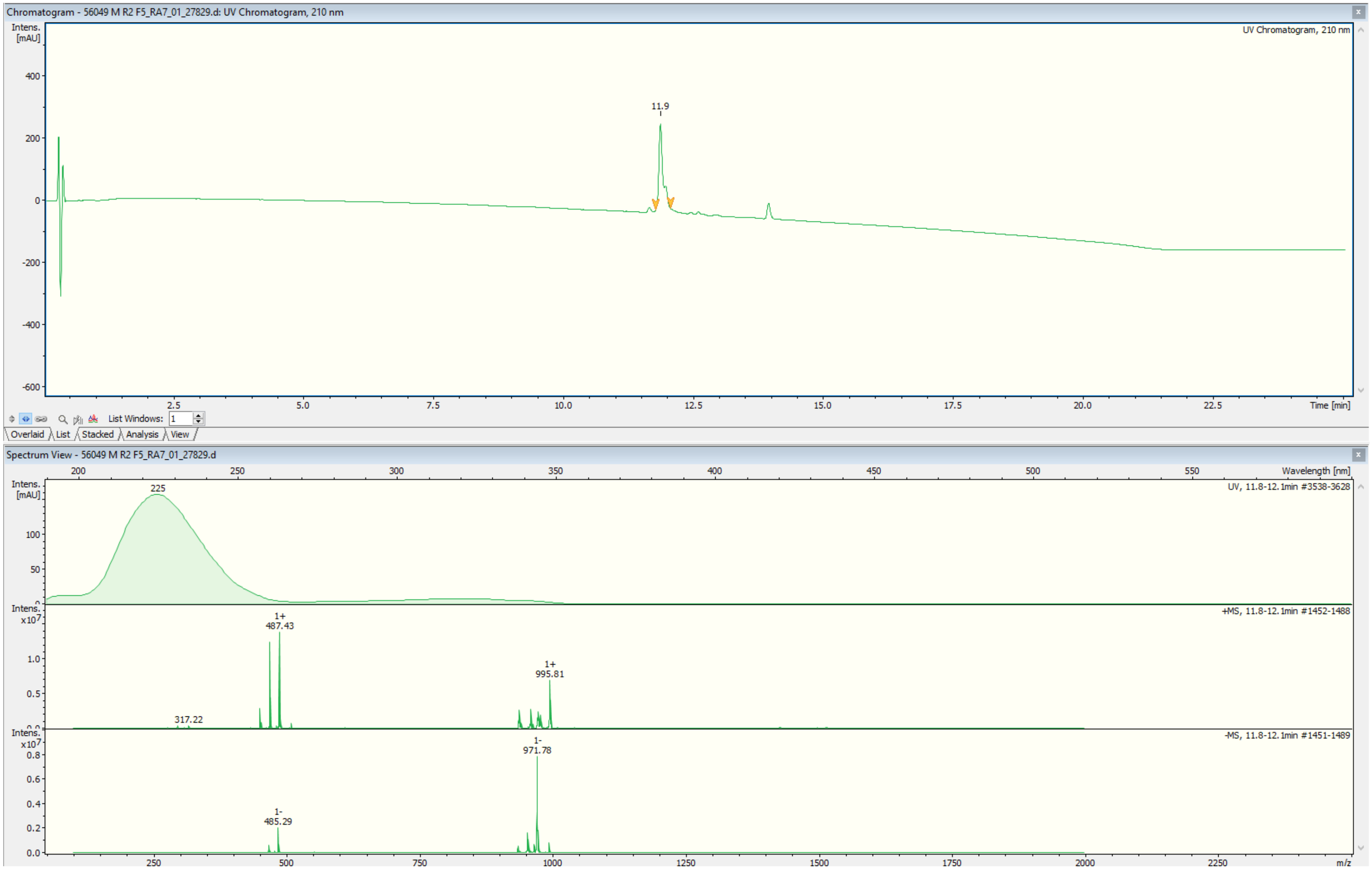

Figure S1: ESIMS data for tumulosic acid (**8**).

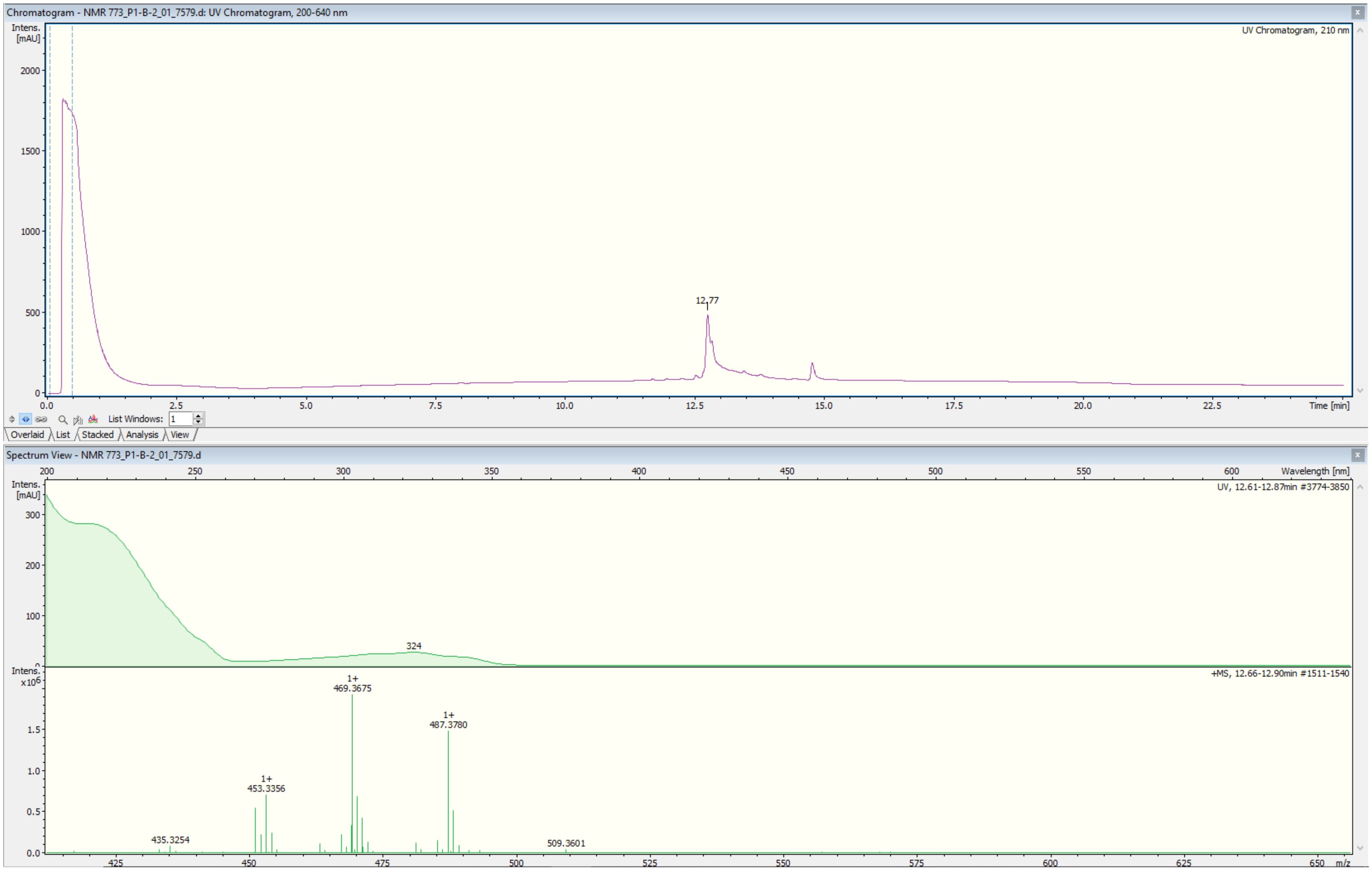

Figure S2: HR-ESIMS data for tumulosic acid (**8**).

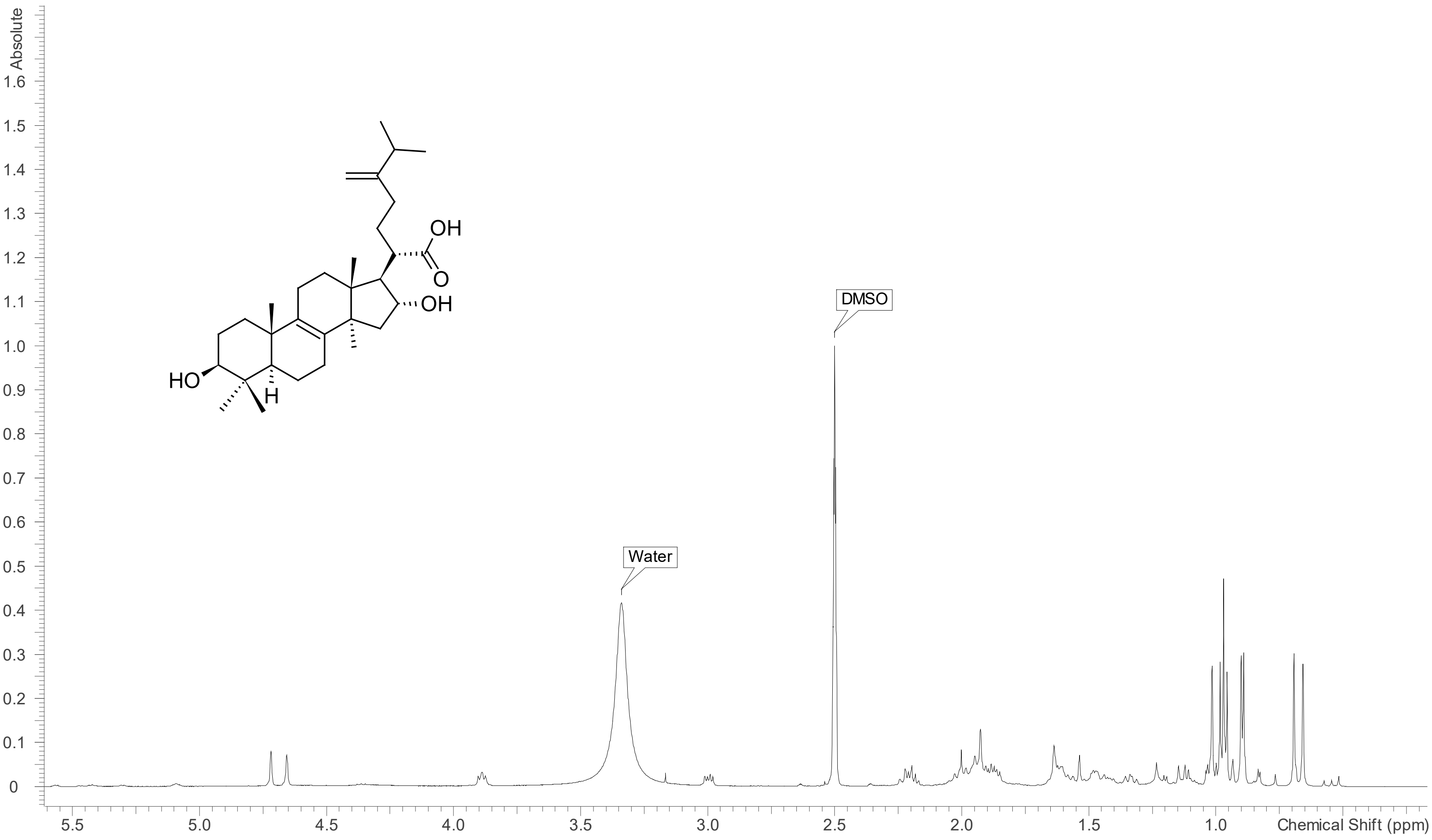

Figure S3: ^{1}H NMR spectrum (DMSO-d_6, 700 MHz) of tumulosic acid (**8**).

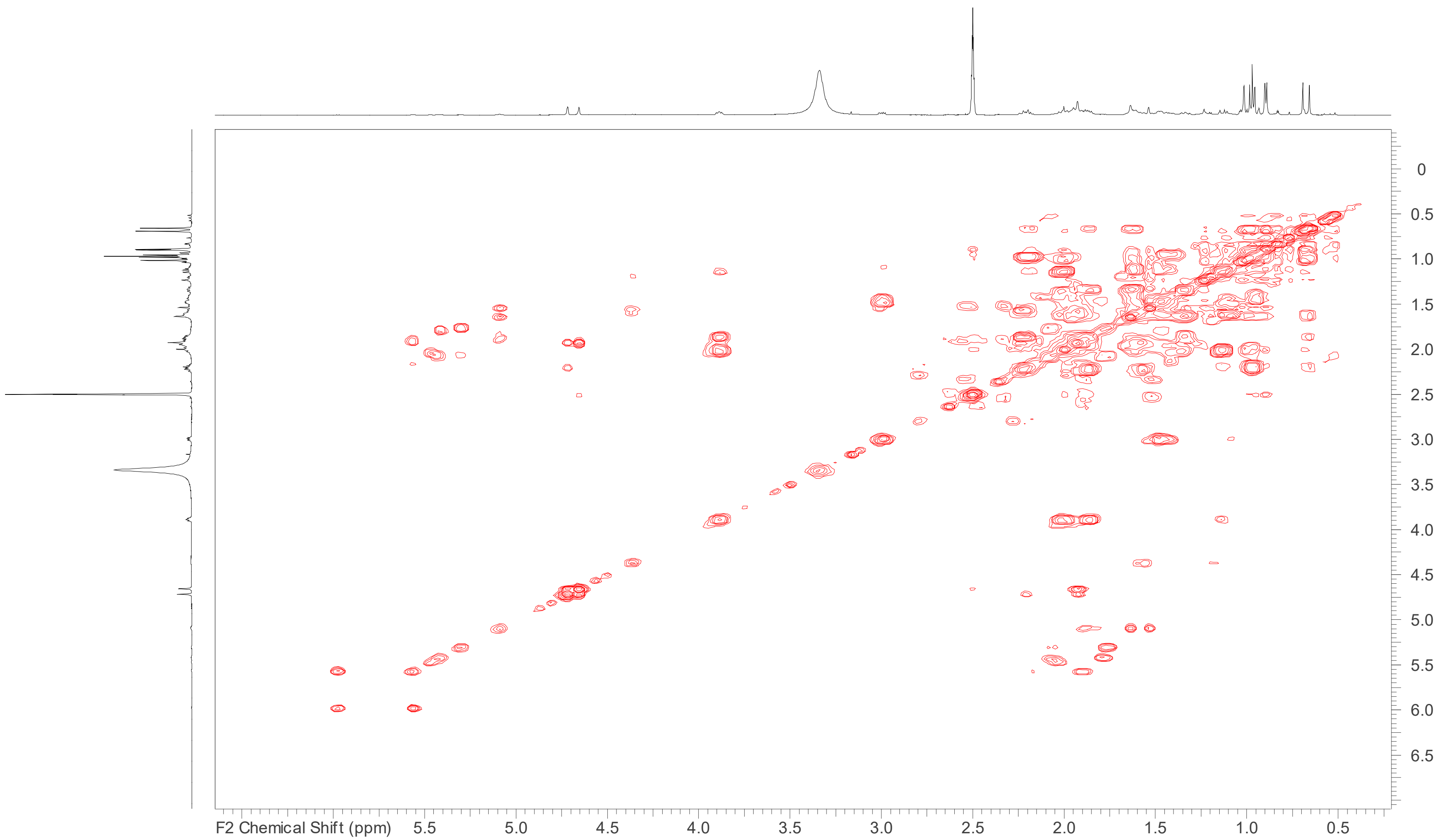

Figure S4: COSY spectrum (DMSO- *d*$_{6}$, 700 MHz) of tumulosic acid (**8**).

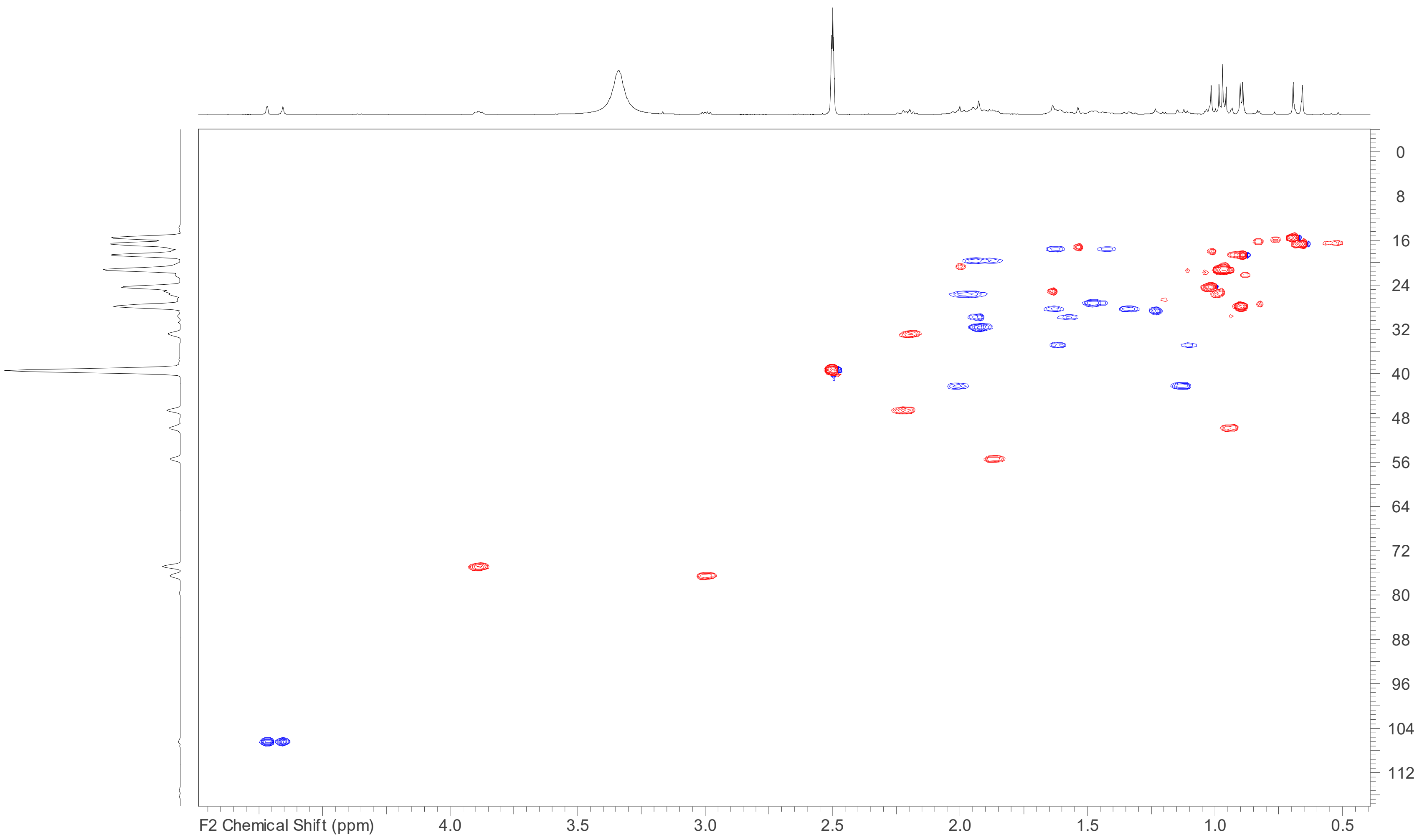

Figure S5: HSQC spectrum (DMSO- d_6, 700 MHz) of tumulosic acid (**8**).

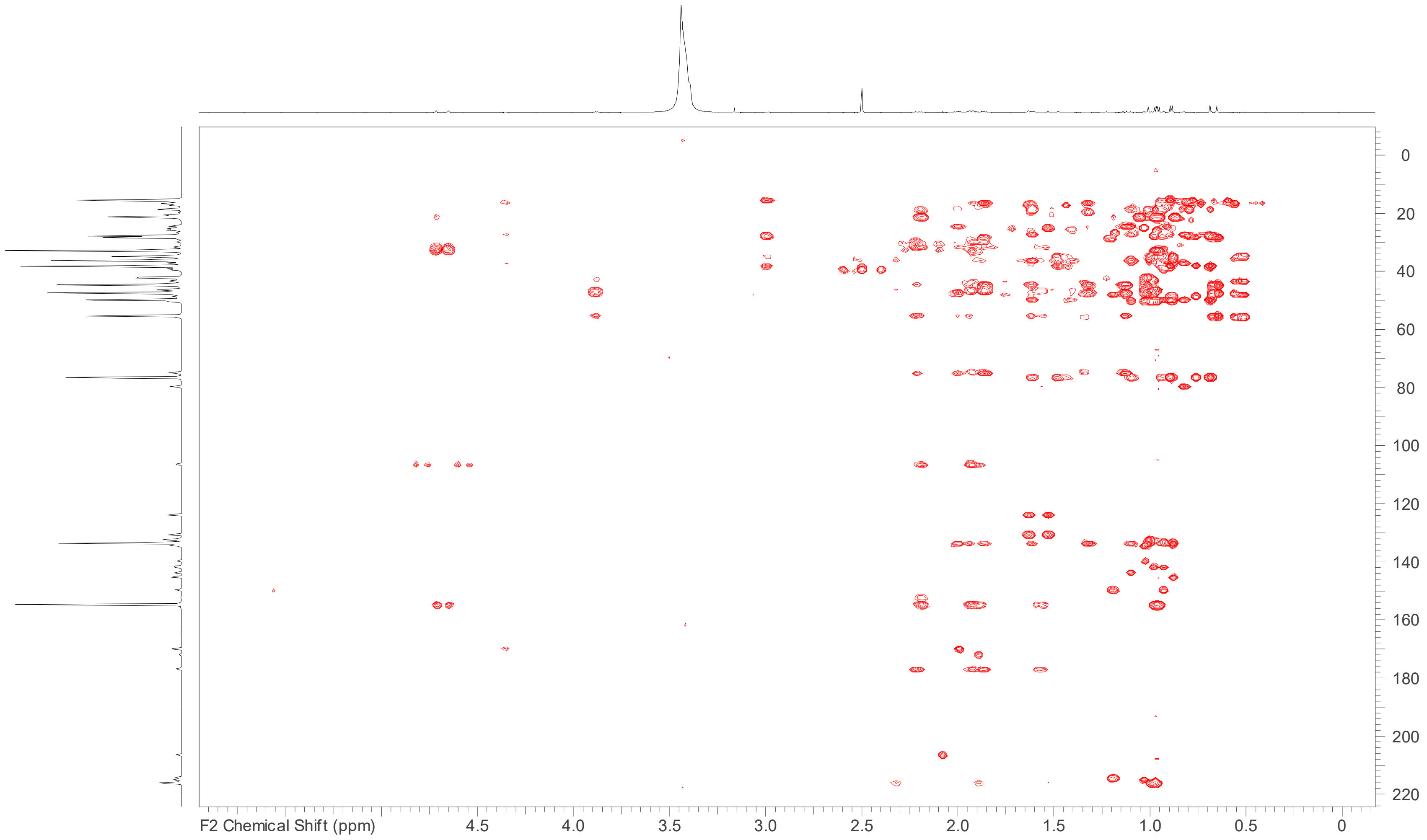

Figure S6: HMBC spectrum (DMSO-d_6, 700 MHz) of tumulosic acid (**8**).

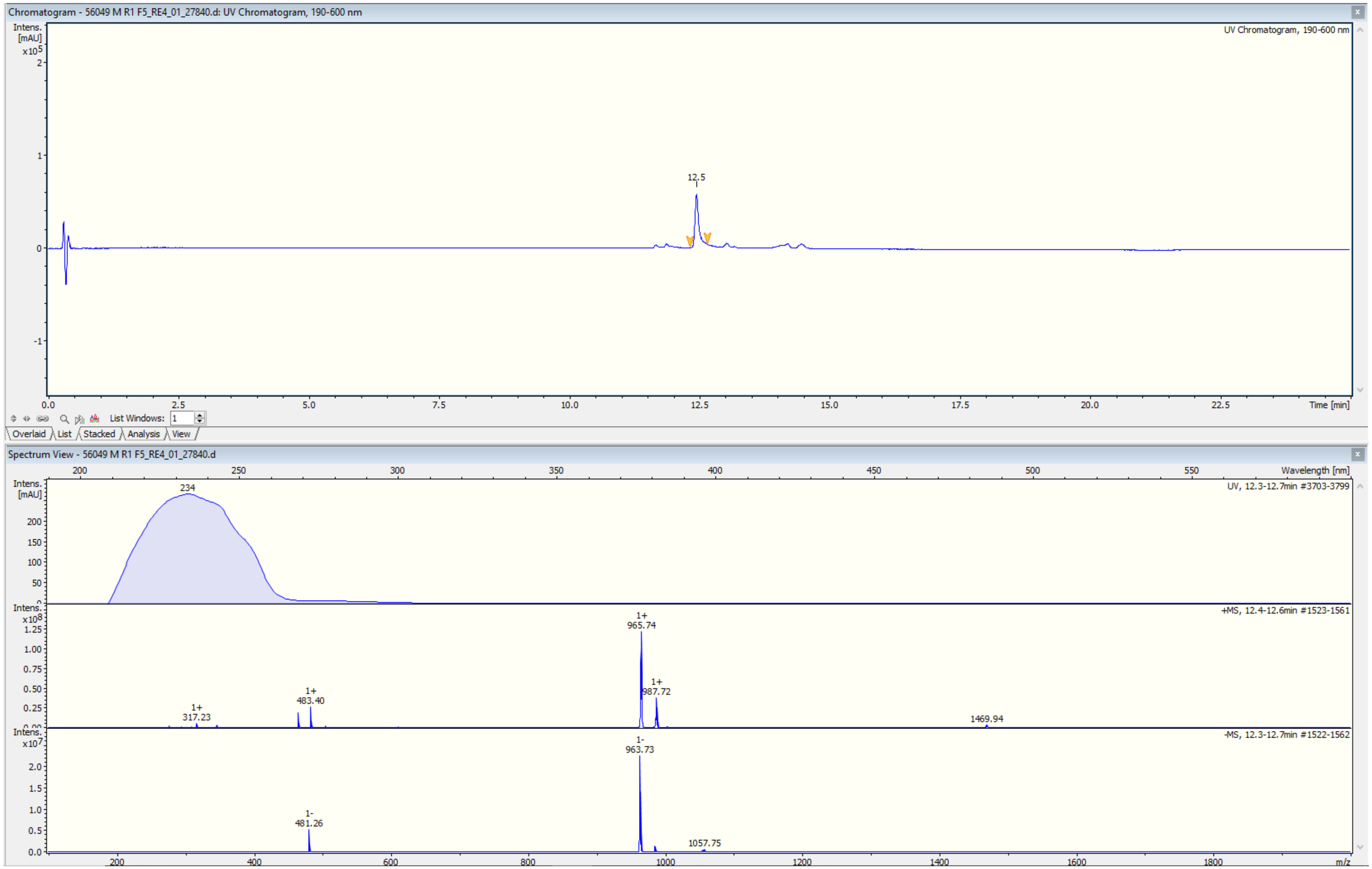

Figure S7: ESIMS data for polyporenic acid C (**9**).

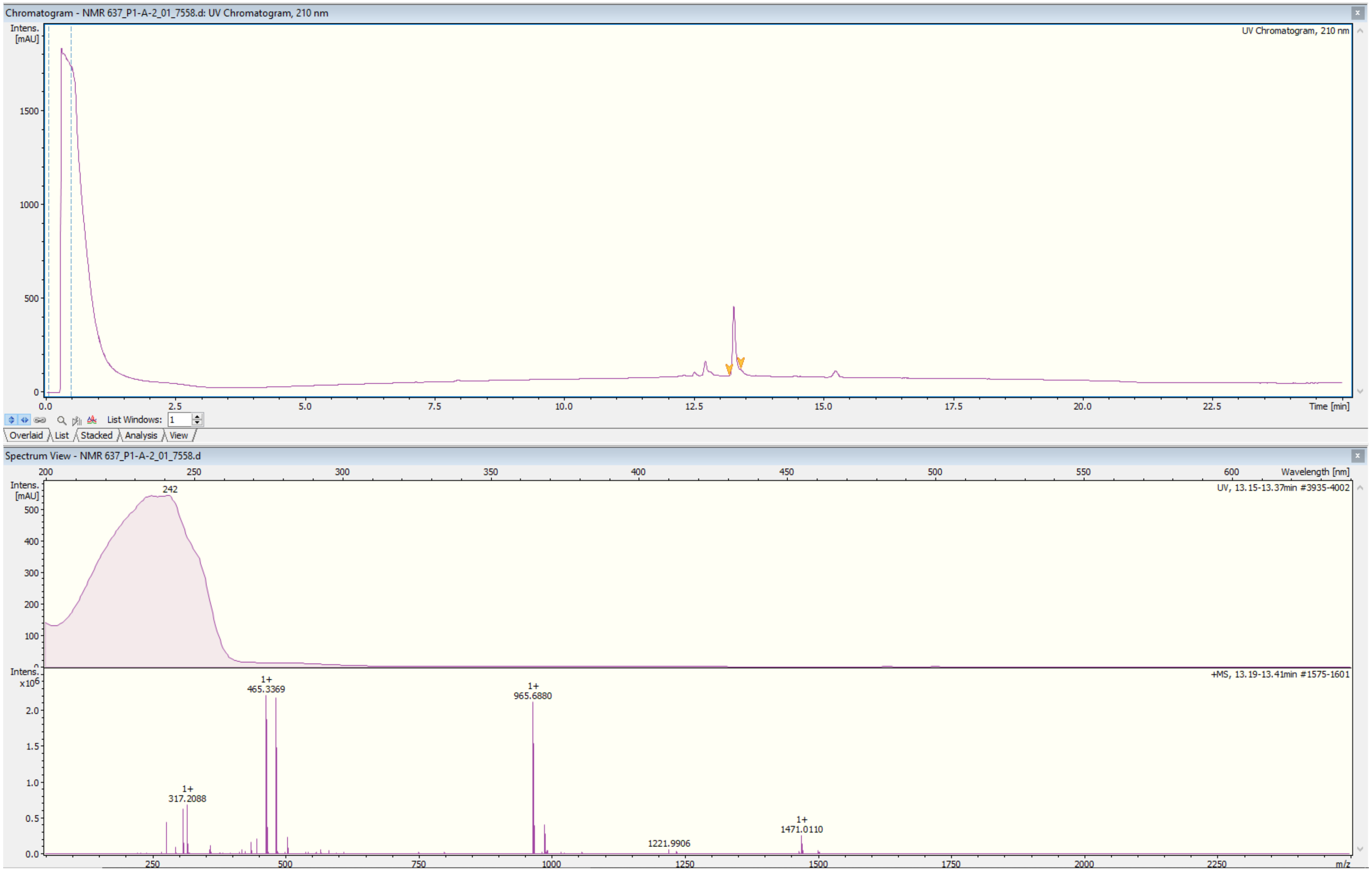

Figure S8: HR-ESIMS data for polyporenic acid C (**9**).

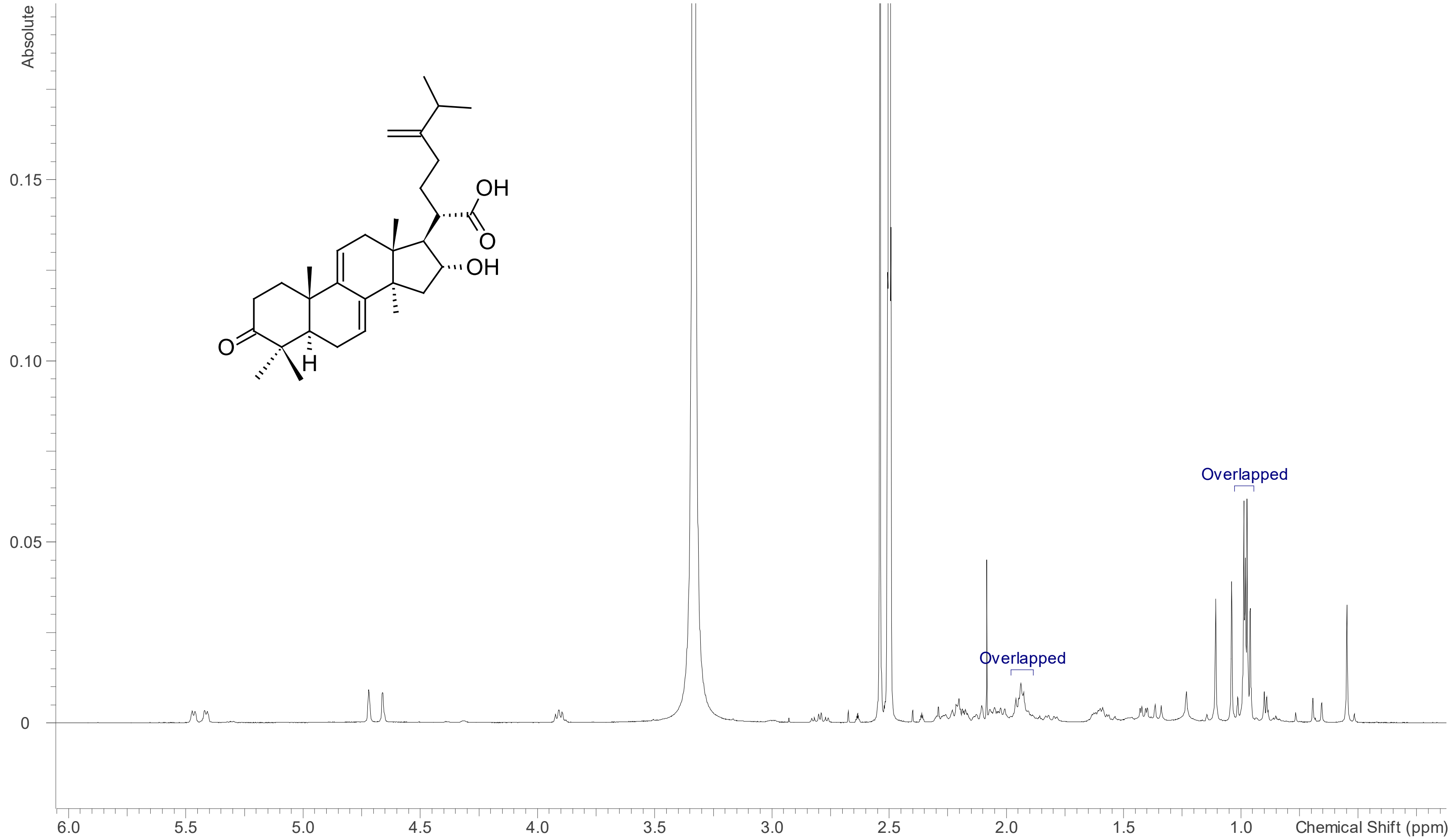

Figure S9: ^{1}H NMR spectrum (DMSO-d_6, 700 MHz) of polyporenic acid C (**9**).

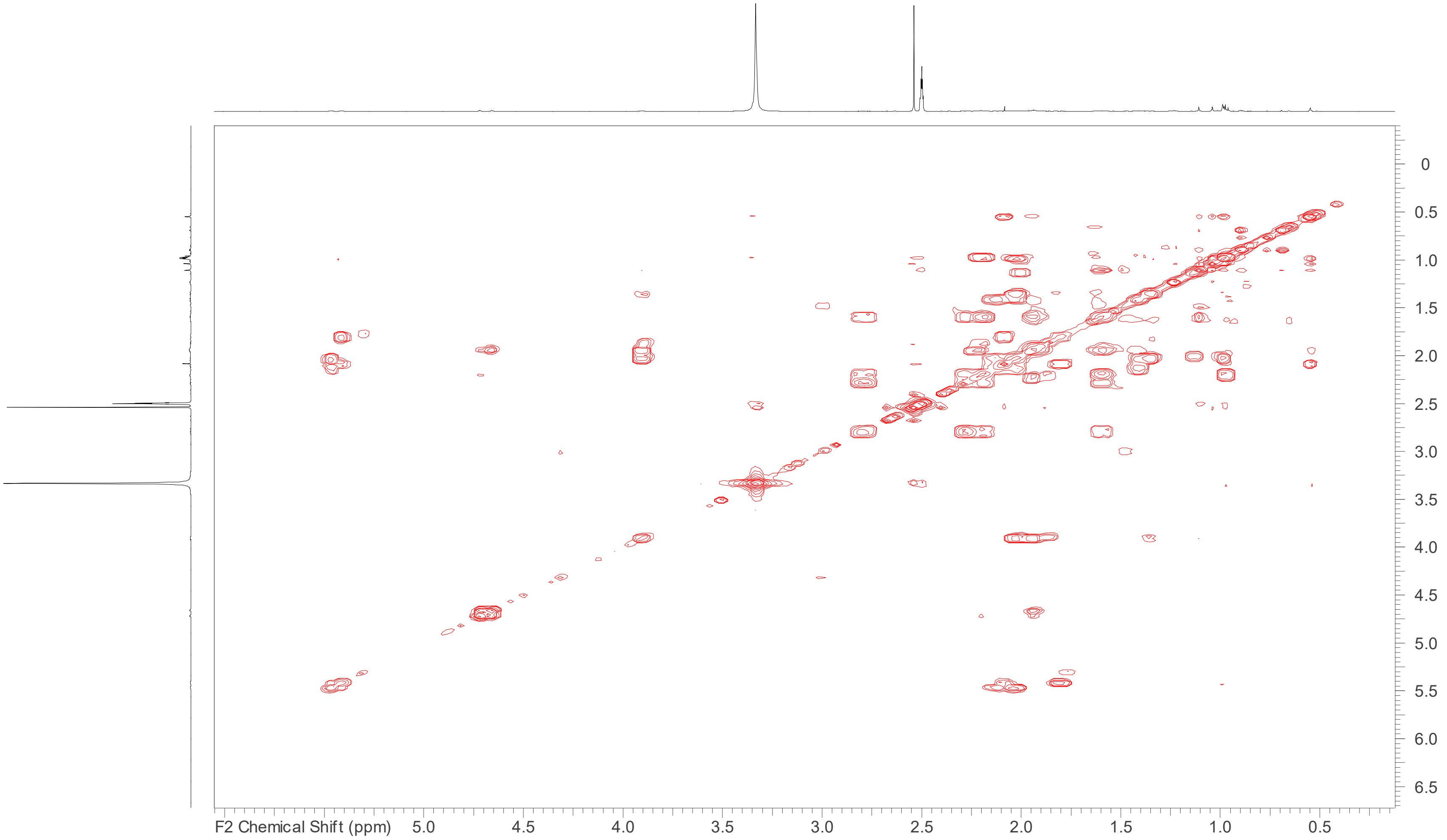

Figure S10: COSY spectrum (DMSO-d_6, 700 MHz) of polyporenic acid C (**9**).

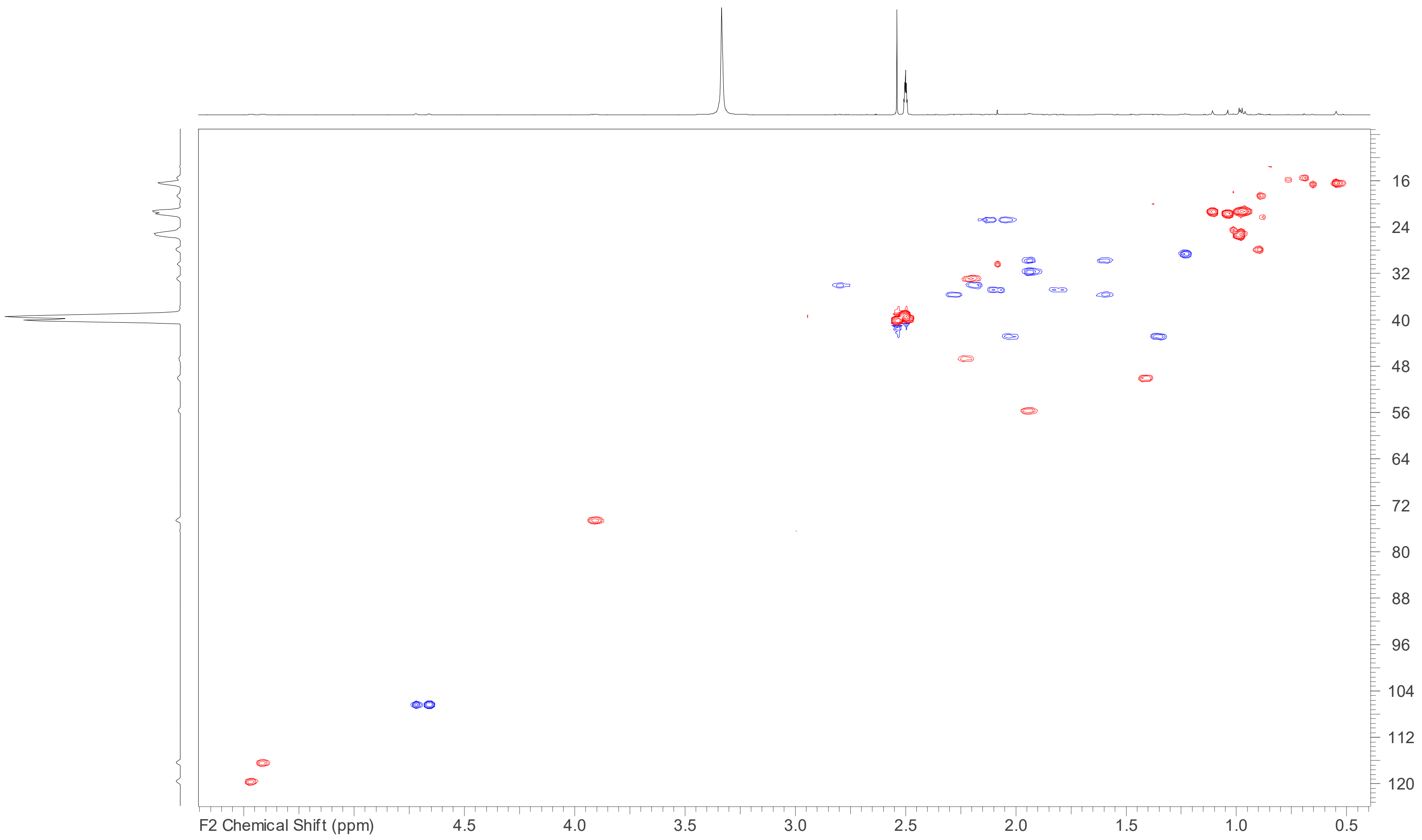

Figure S11: HSQC spectrum (DMSO-d_6, 700 MHz) of polyporenic acid C (**9**).

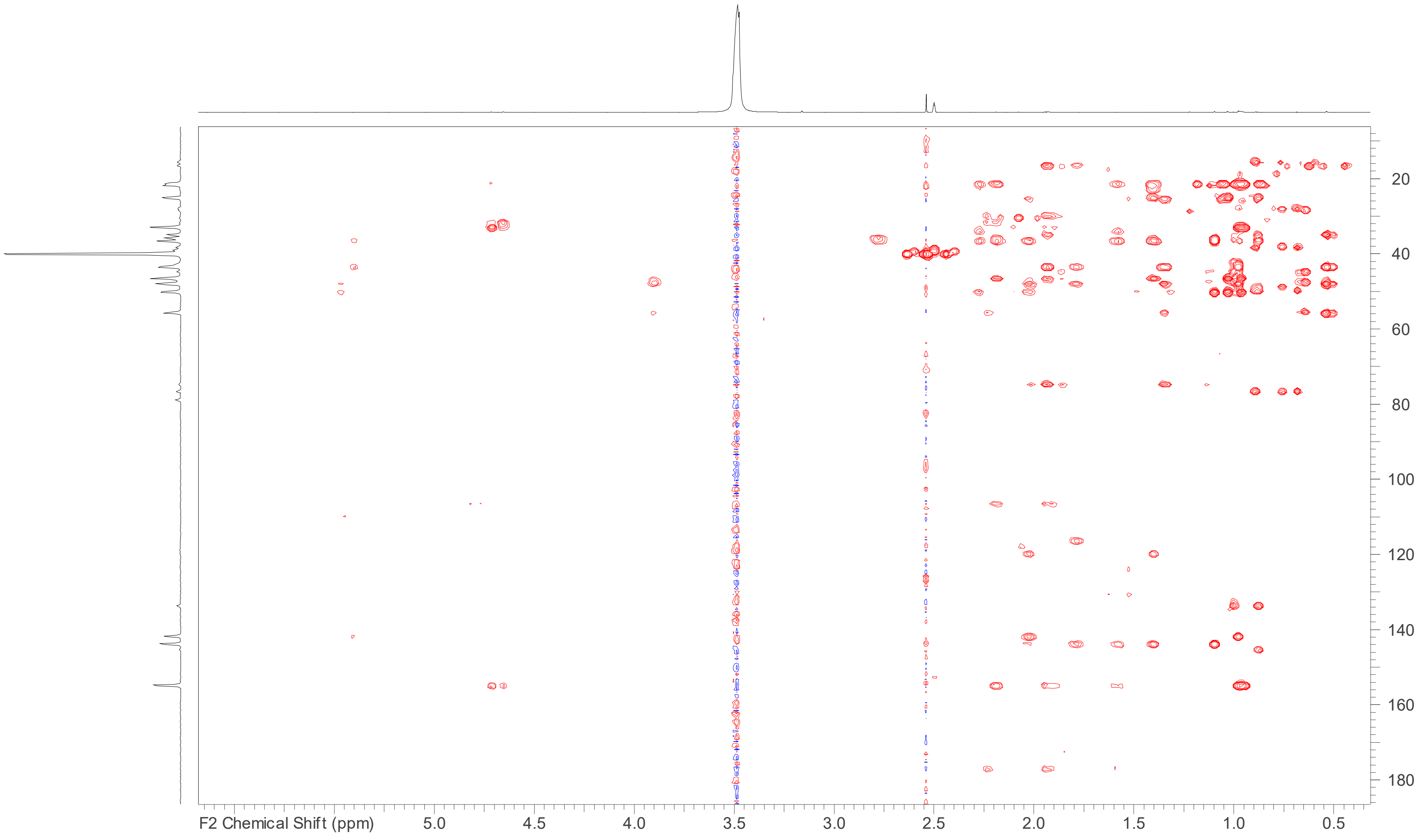

Figure S12: HMBC spectrum (DMSO-d_6, 700 MHz) of polyporenic acid C (**9**).

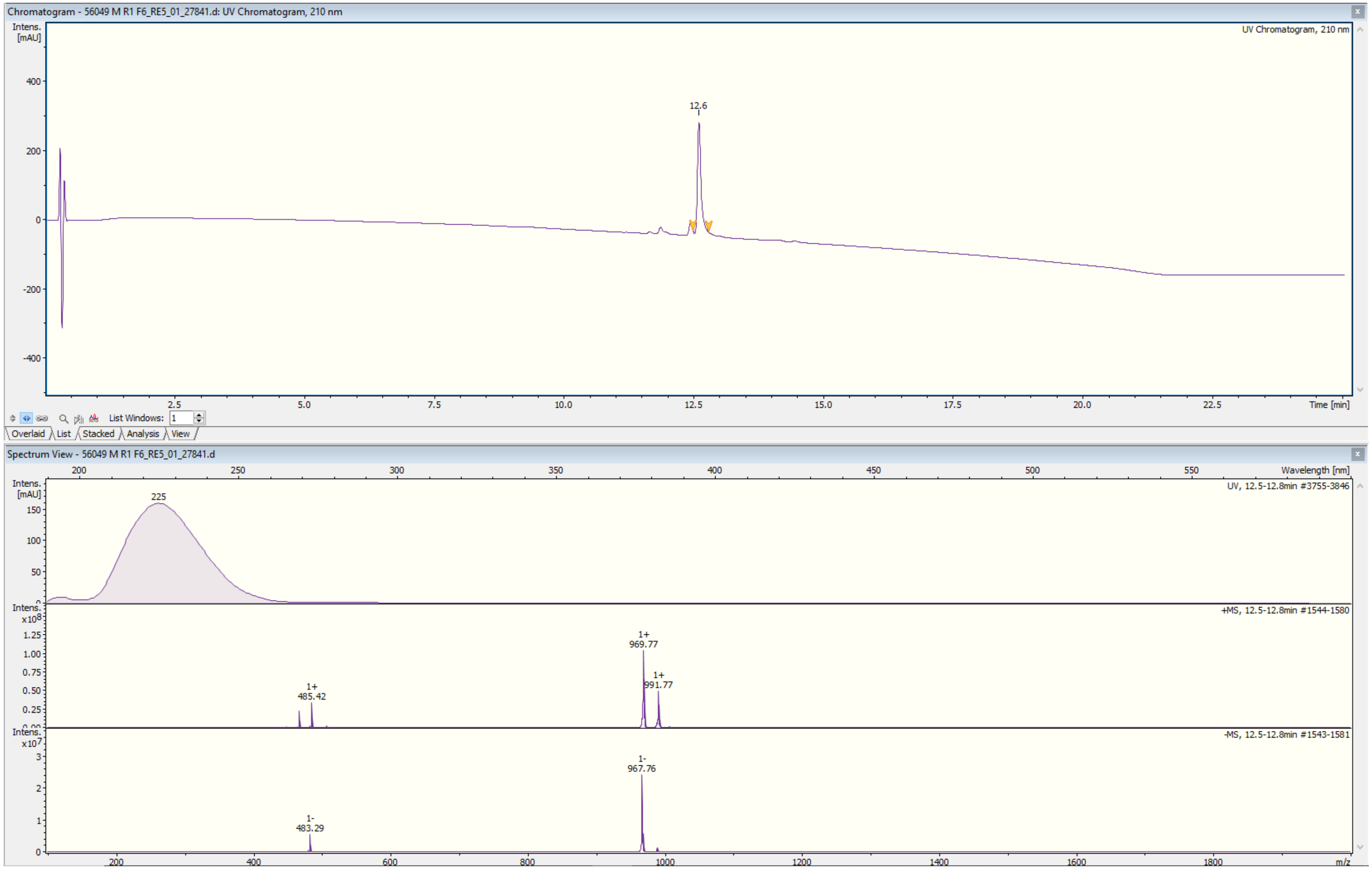

Figure S13: ESIMS data for 16α-hydroxyeburiconic acid (**10**).

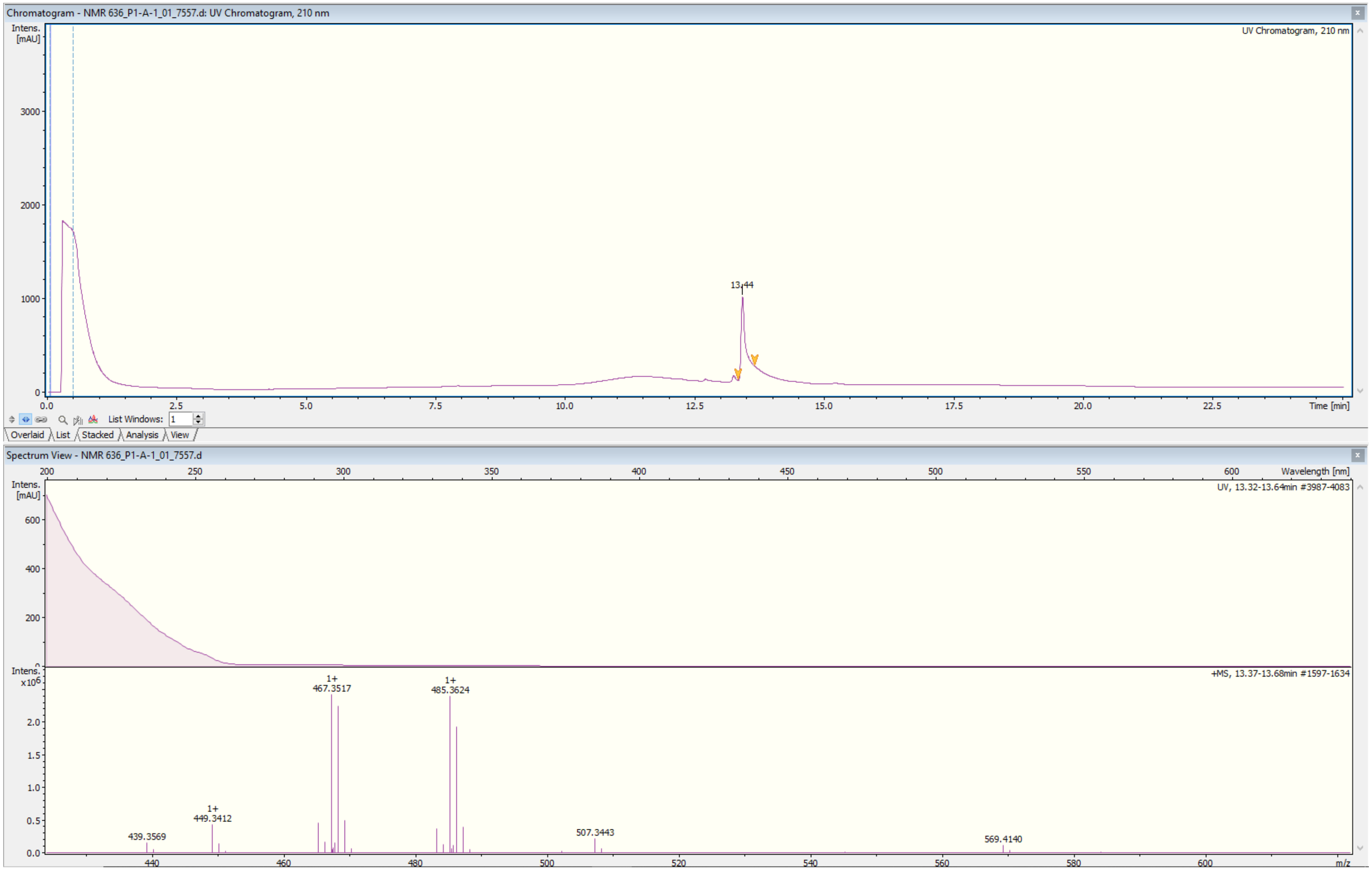

Figure S14: HR-ESIMS data for 16α-hydroxyeburiconic acid (**10**).

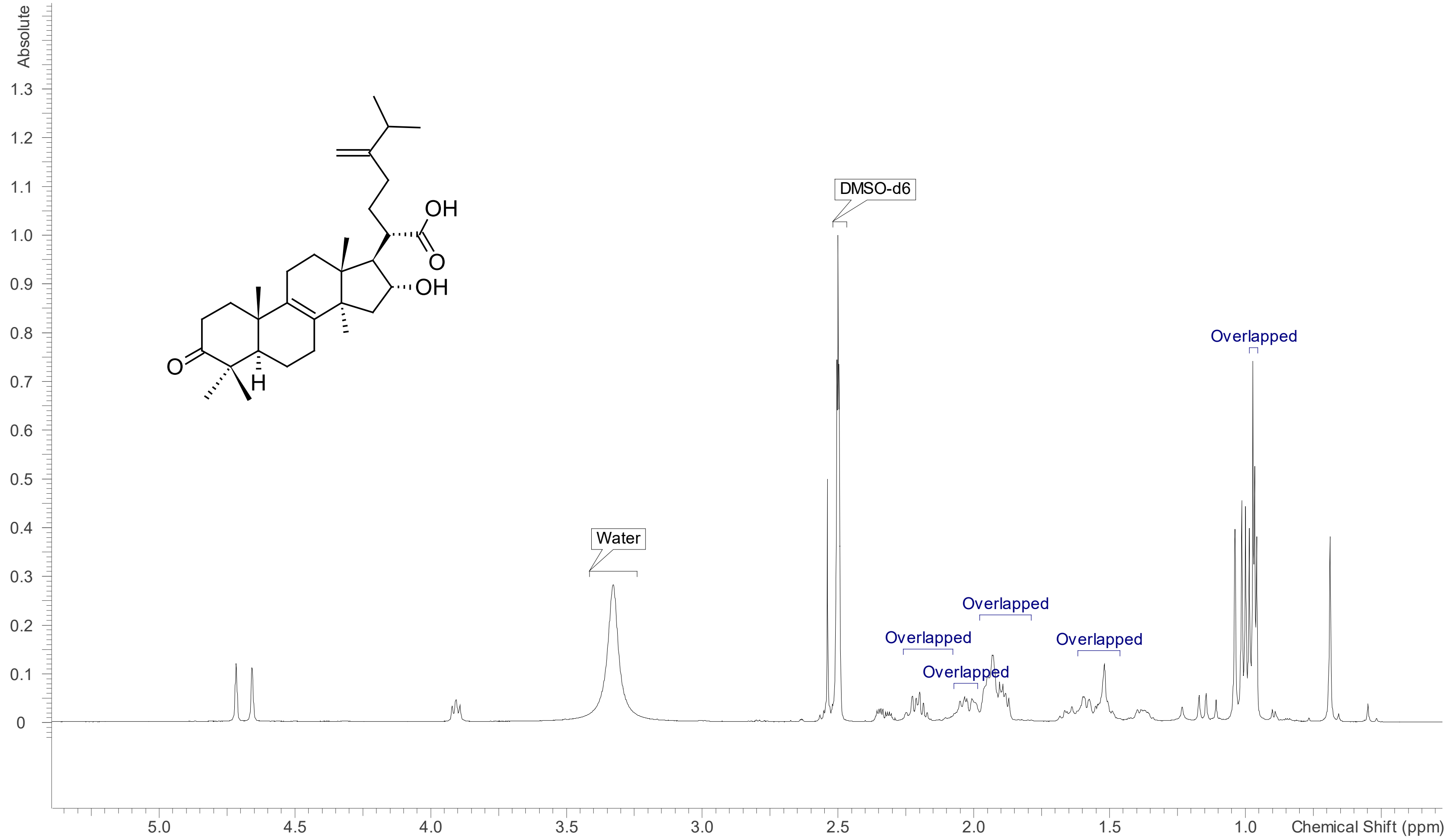

Figure S15: ^{1}H NMR spectrum (DMSO-d_6, 700 MHz) of 16α-hydroxyeburiconic acid (**10**).

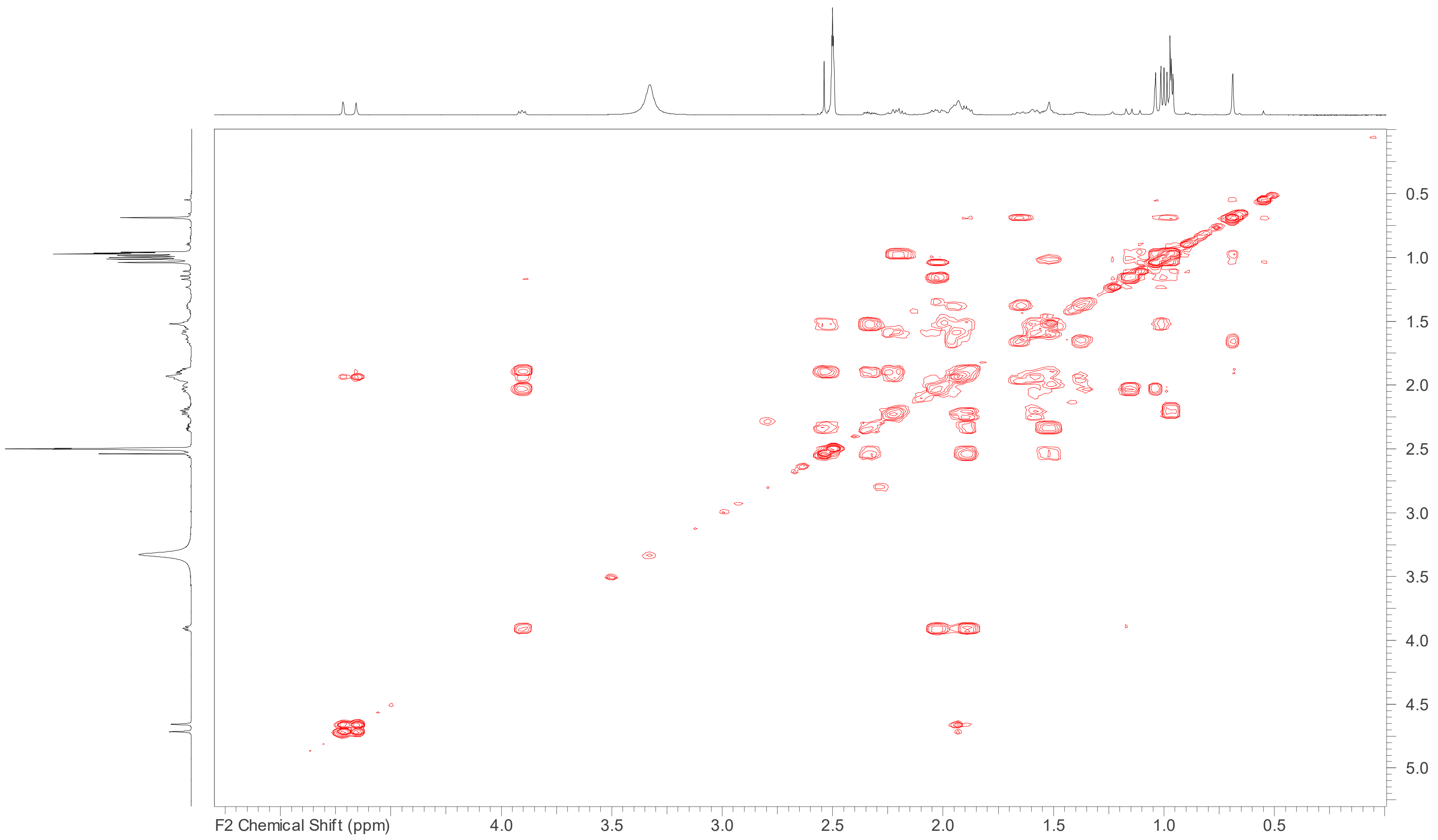

Figure S16: COSY spectrum (DMSO- d_6, 700 MHz) of 16α-hydroxyeburiconic acid (**10**).

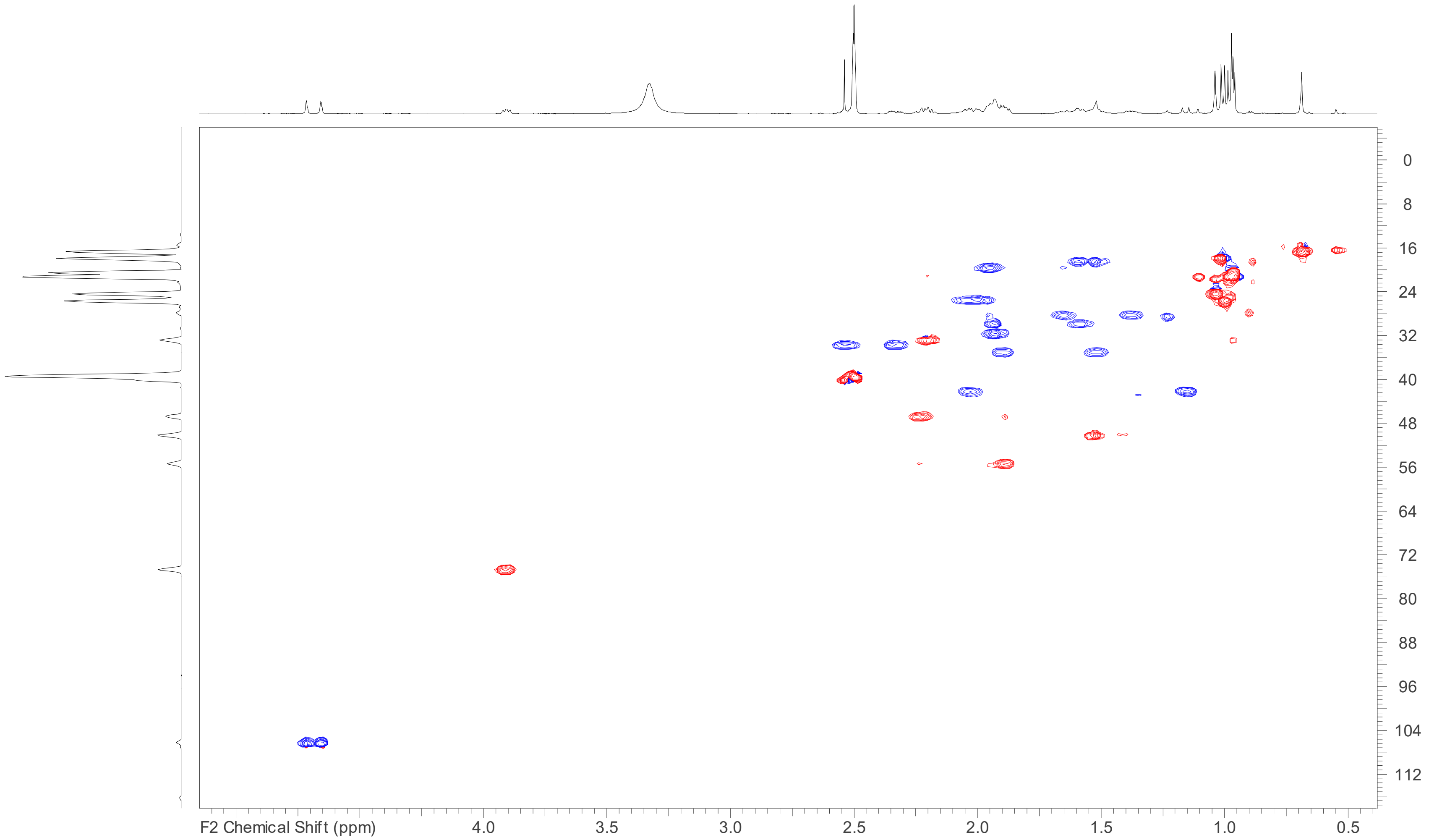

Figure S17: HSQC spectrum (DMSO-d_6, 700 MHz) of 16α-hydroxyeburiconic acid (**10**).

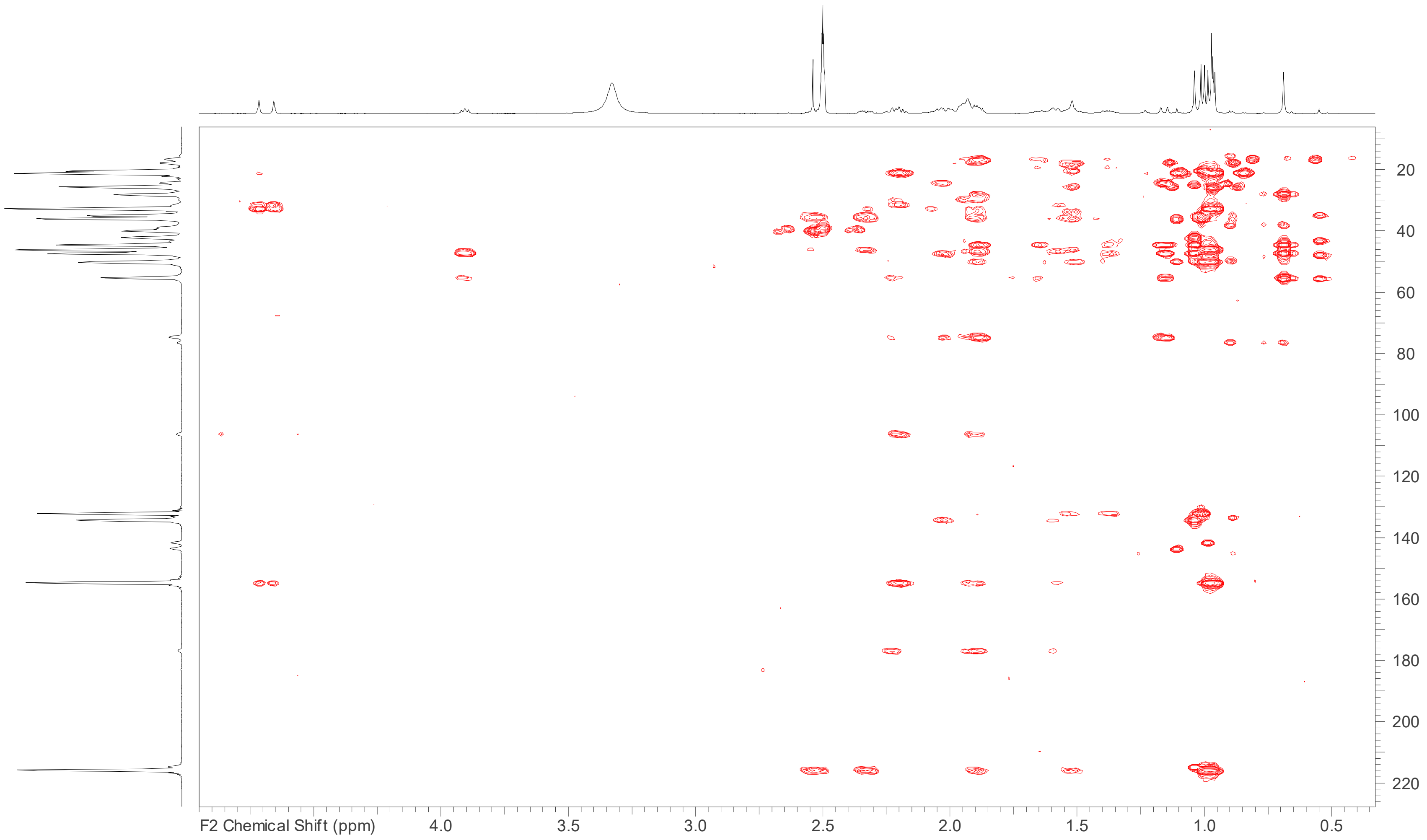

Figure S18: HMBC spectrum (DMSO-d_6, 700 MHz) of 16α-hydroxyeburiconic acid (**10**).

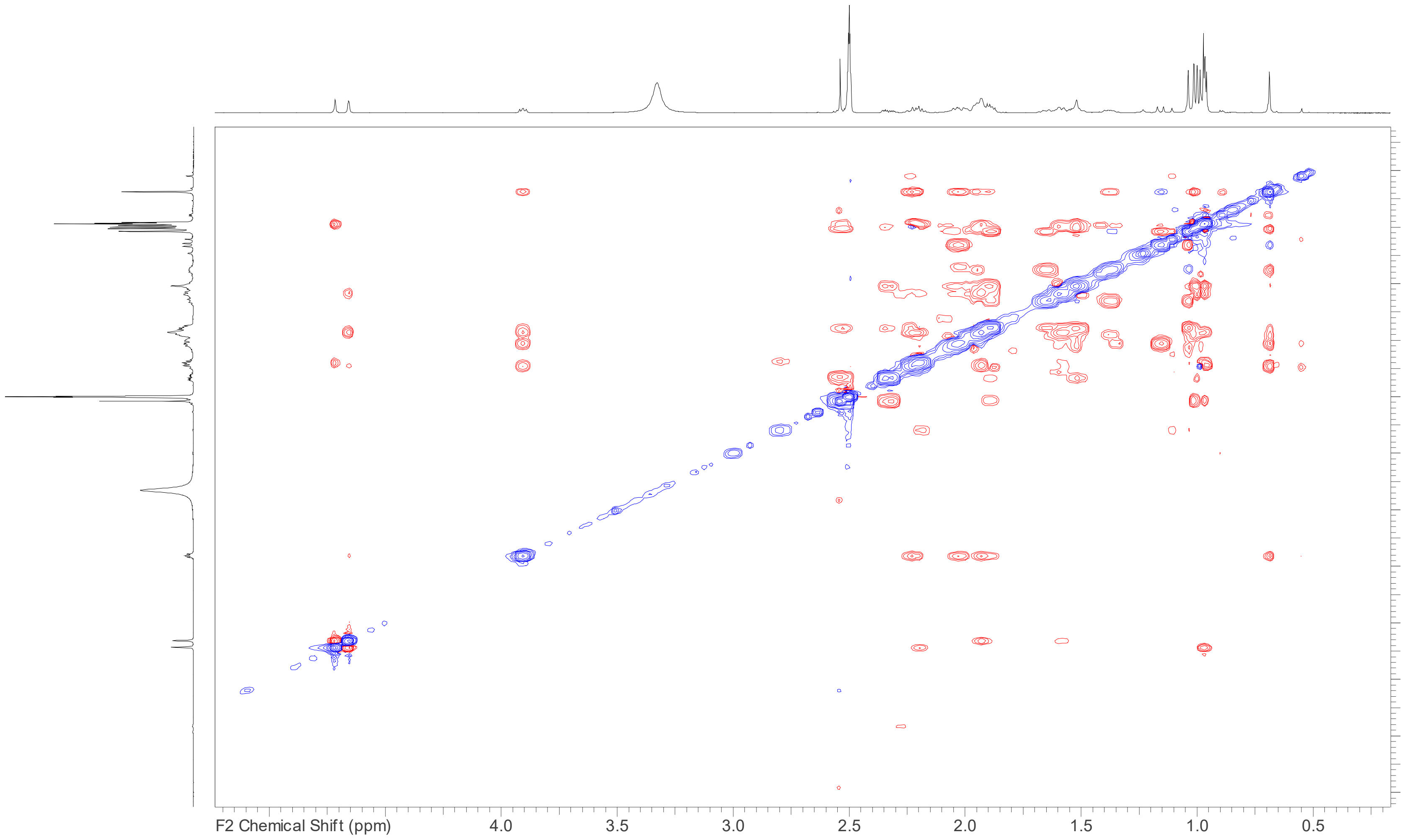

Figure S19: ROESY spectrum (DMSO-d_6, 700 MHz) of 16α-hydroxyeburiconic acid (**10**).

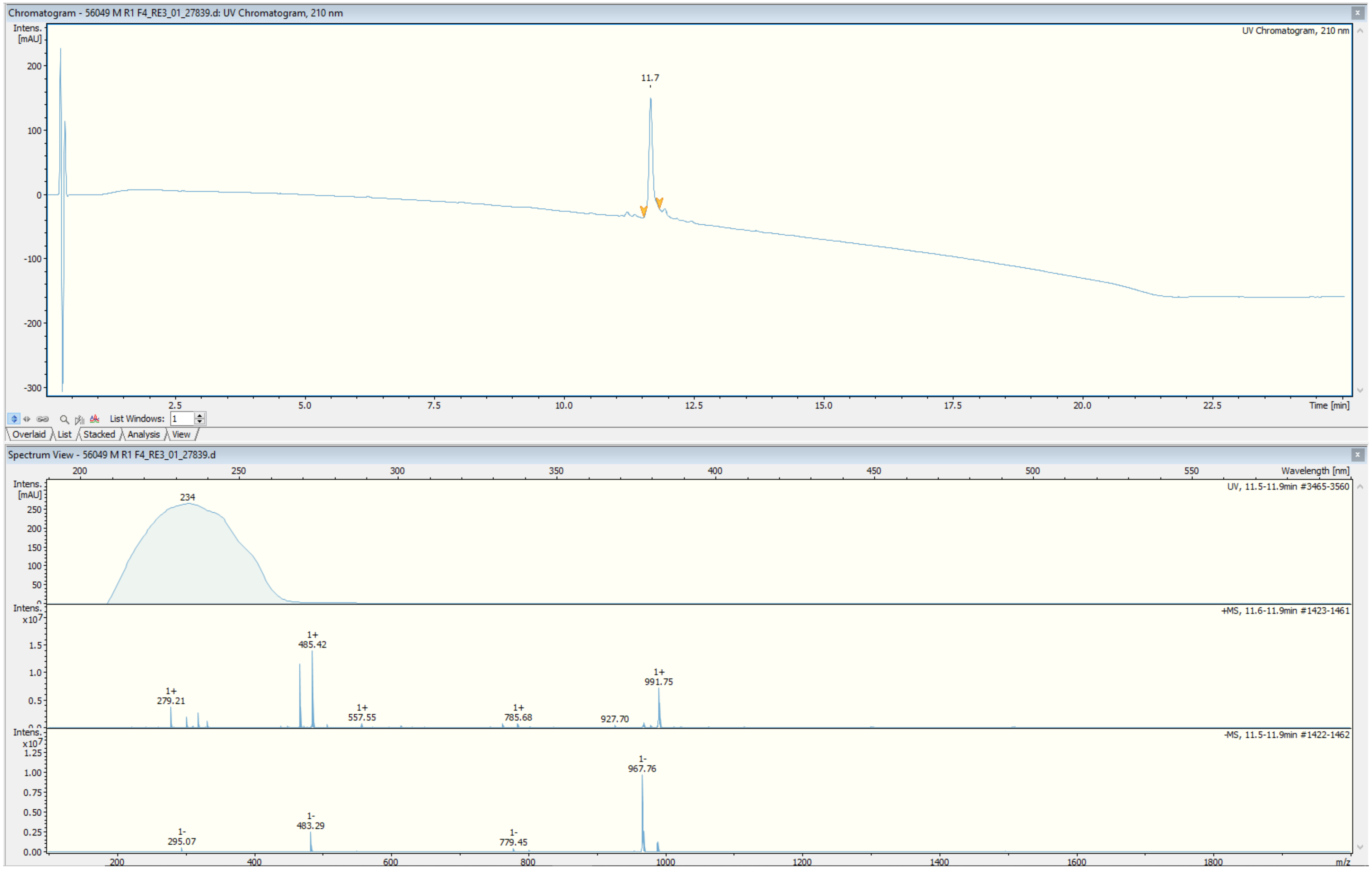

Figure S20: ESIMS data for dehydrotumulosic acid (**11**)

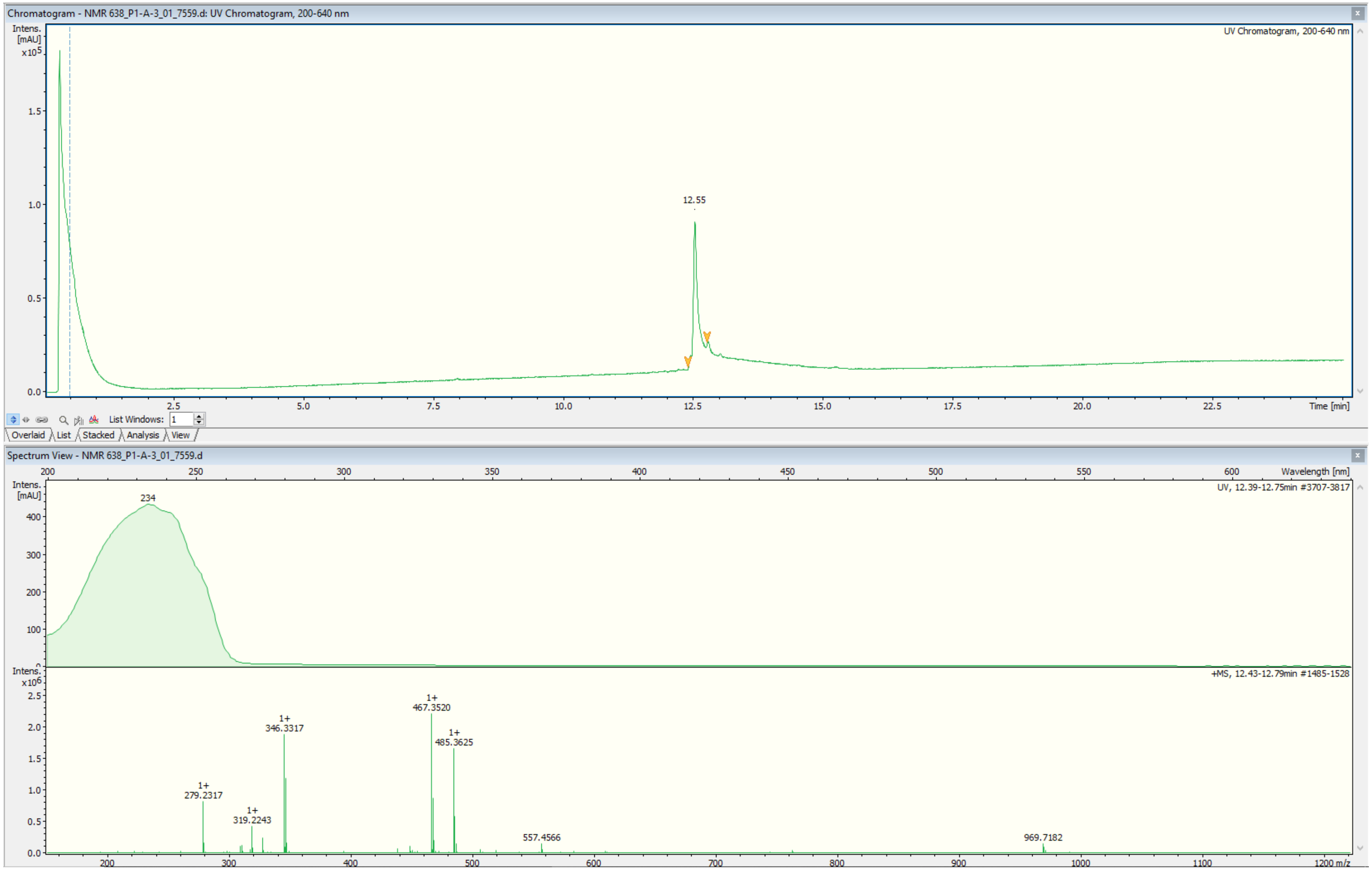

Figure S21: HR-ESIMS data for dehydrotumulosic acid (**11**).

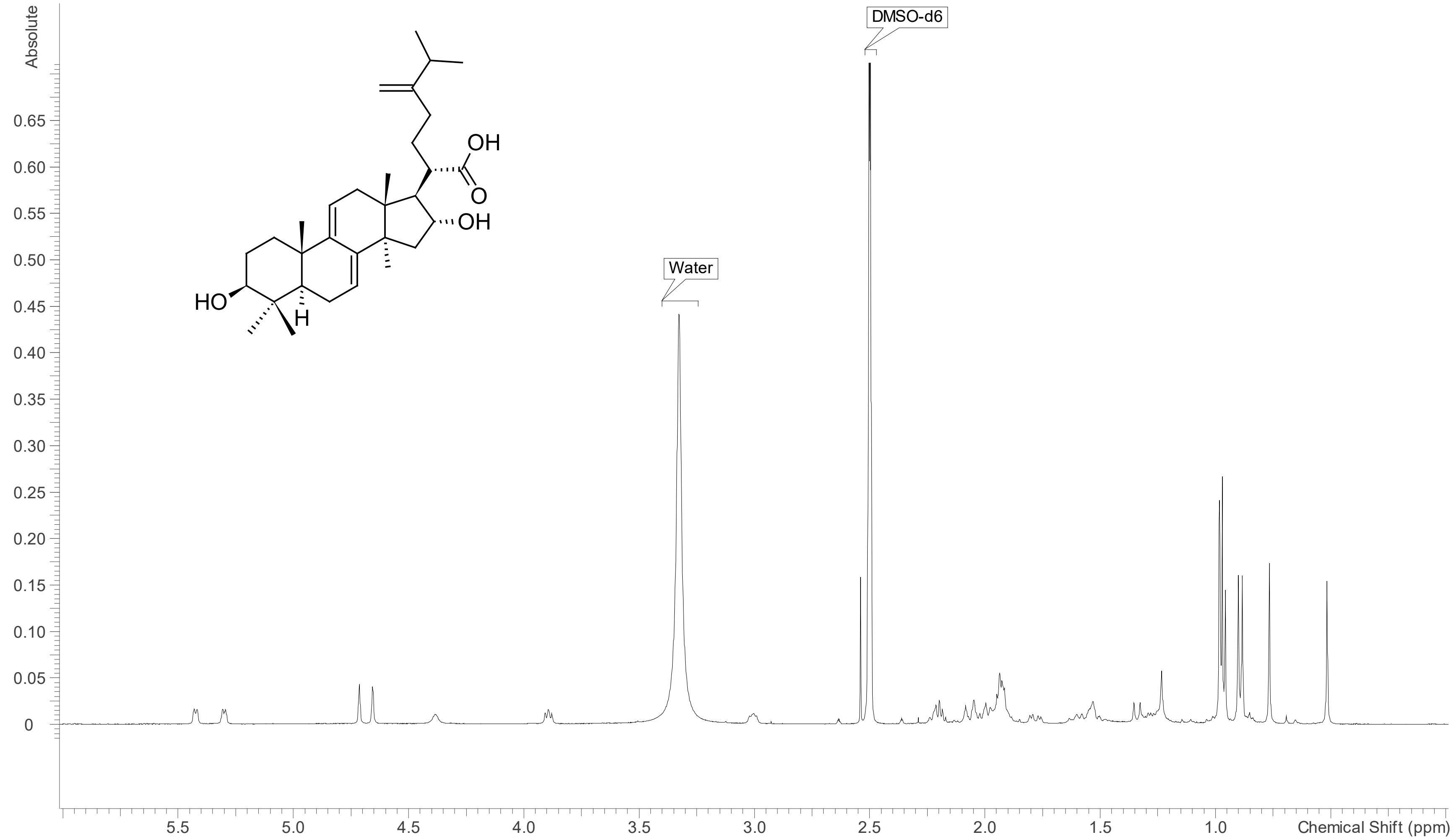

Figure S22: ^{1}H NMR spectrum (DMSO-d_6, 700 MHz) of dehydrotumulosic acid (**11**).

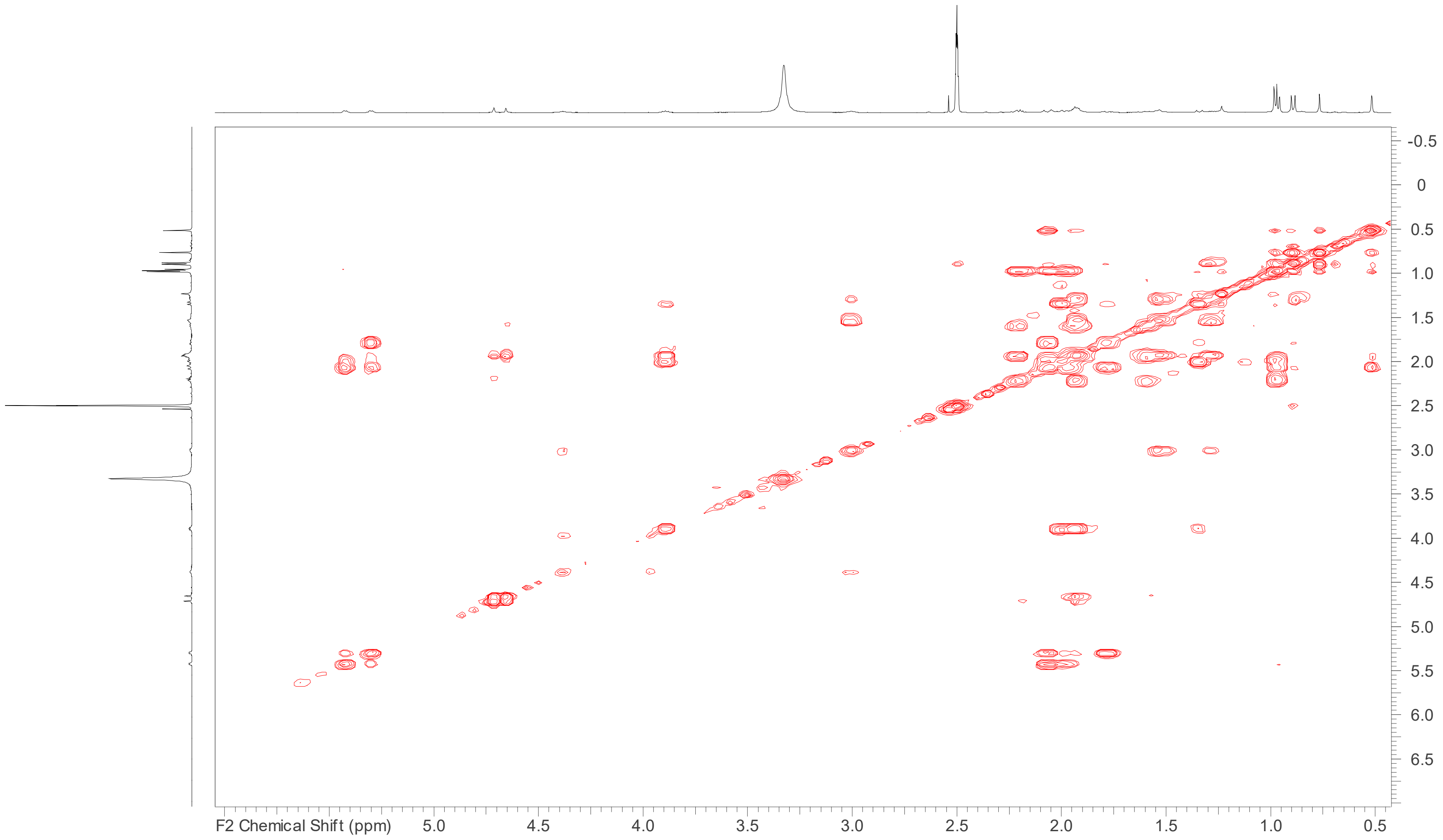

Figure S23: COSY spectrum (DMSO-d_6, 700 MHz) of dehydrotumulosic acid (**11**).

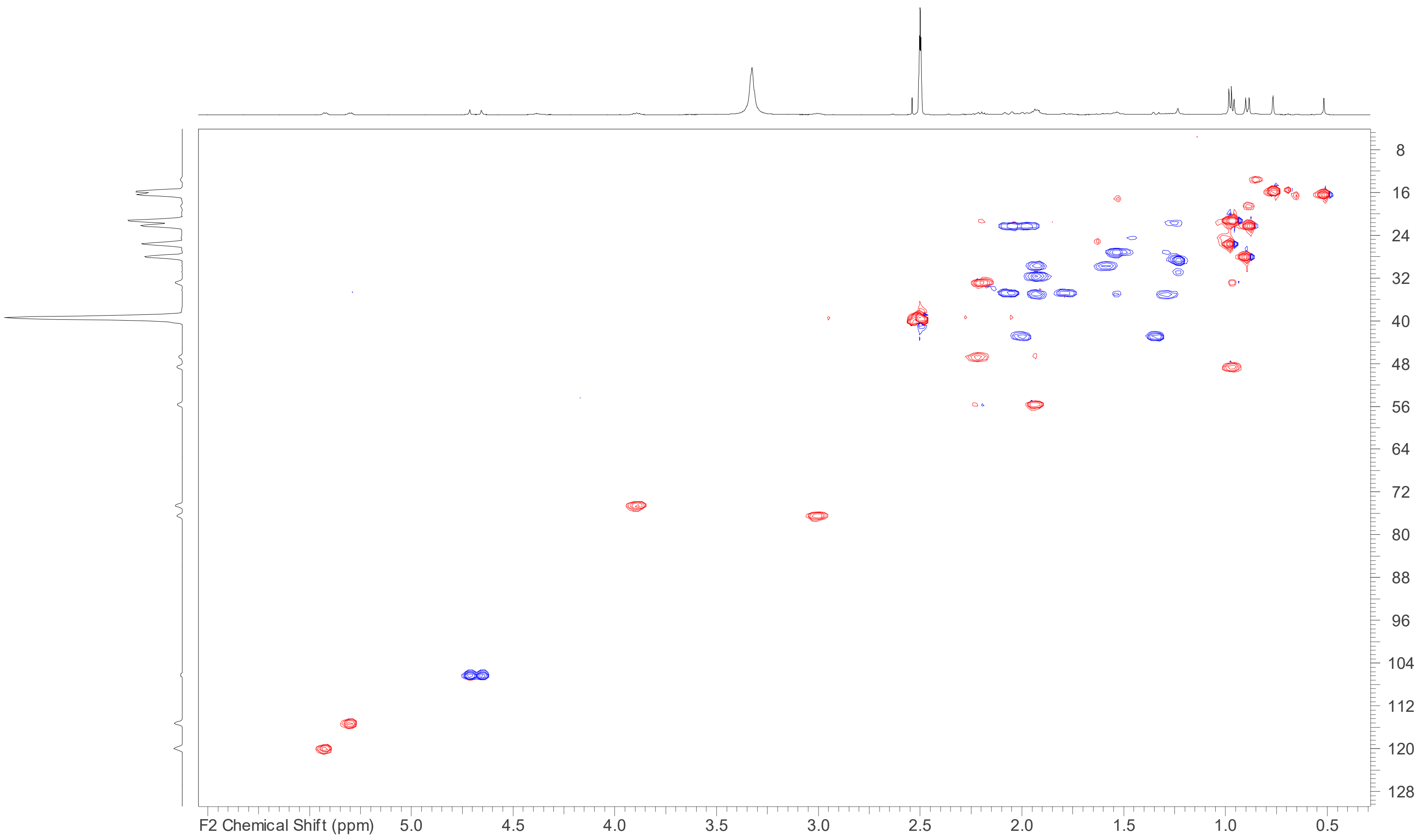

Figure S24: HSQC spectrum (DMSO-d_6, 700 MHz) of dehydrotumulosic acid (**11**).

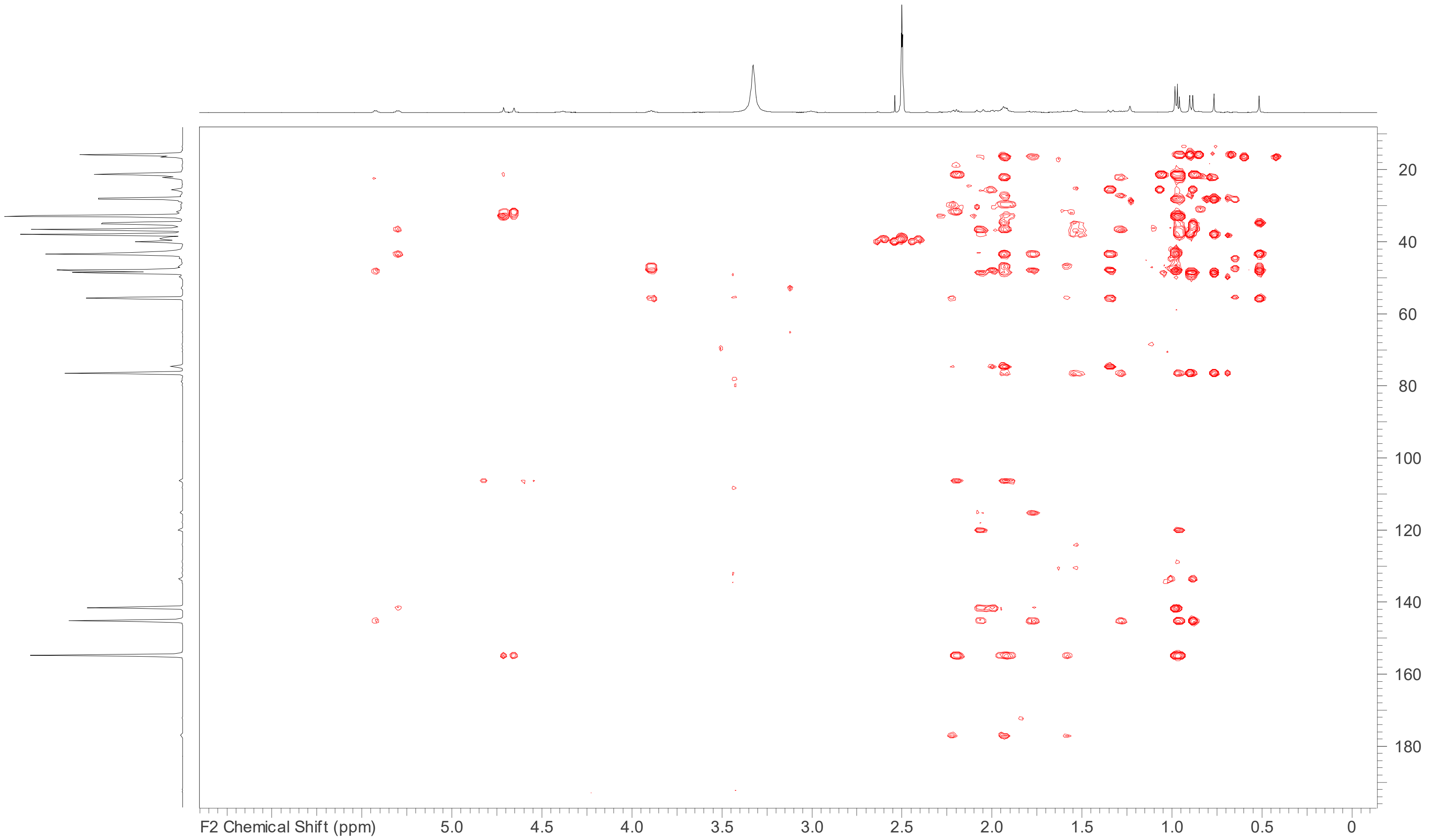

Figure S25: HMBC spectrum (DMSO-d_6, 700 MHz) of dehydrotumulosic acid (**11**).

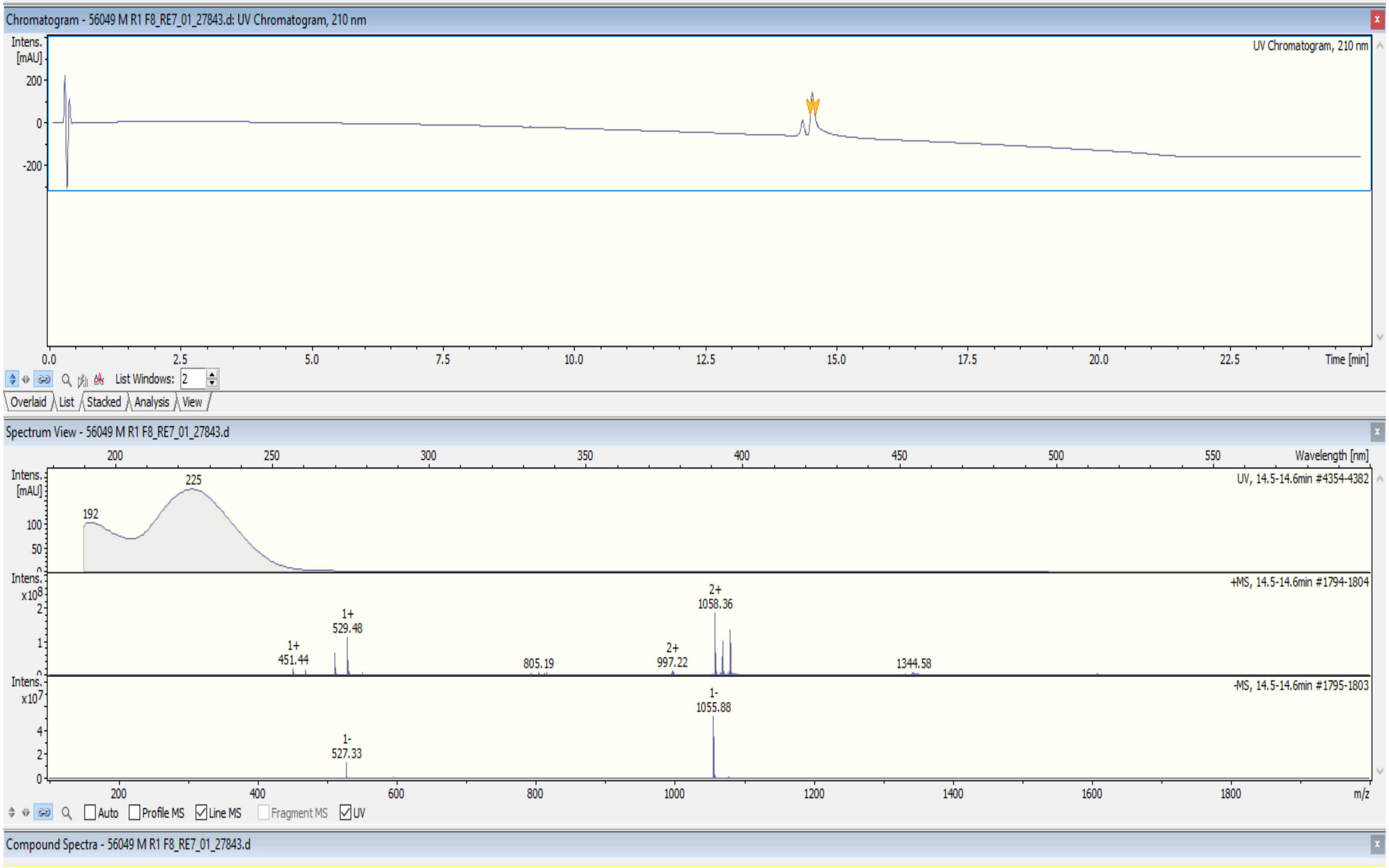

Figure S26: ESIMS data for pachymic acid (**12**)

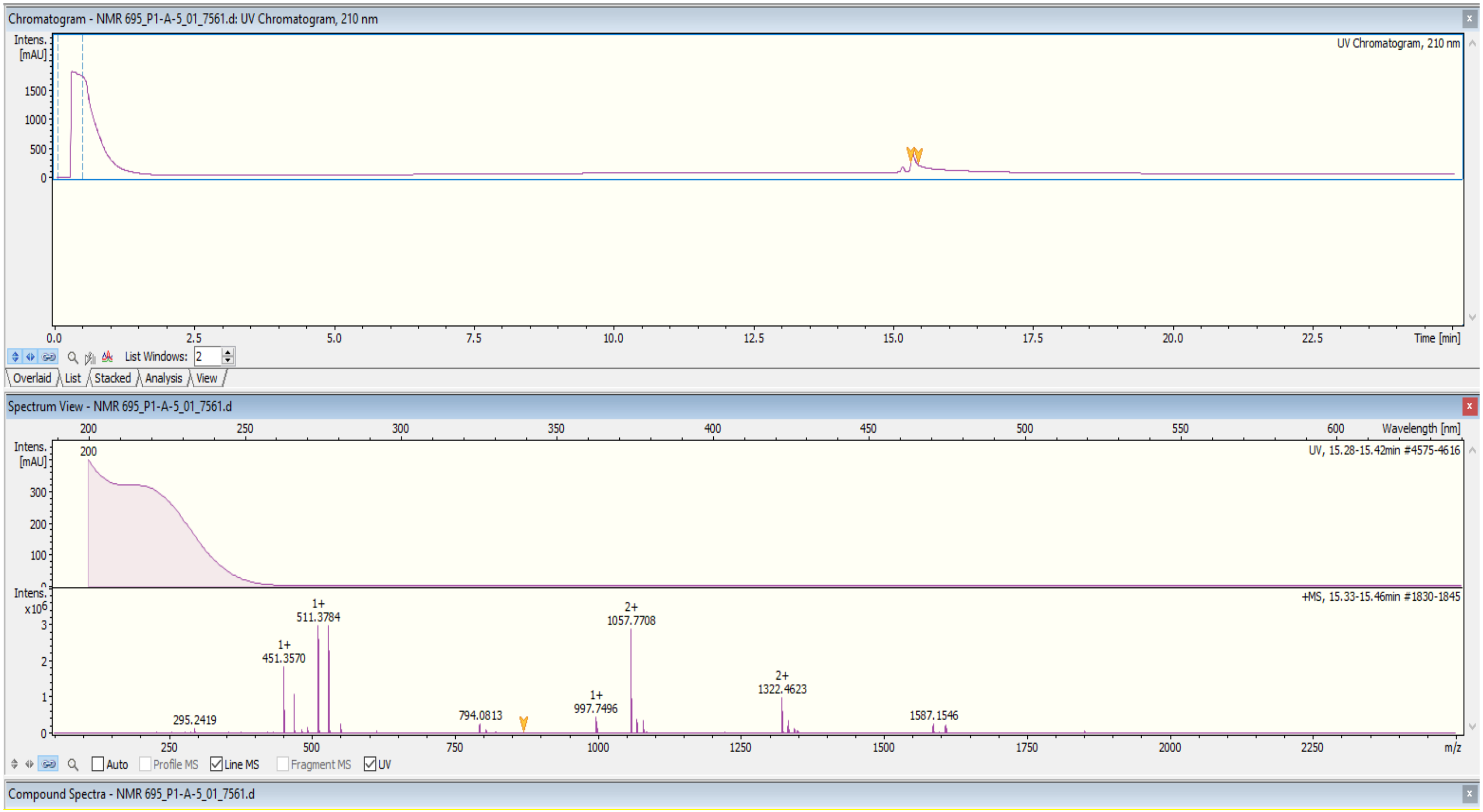

Figure S27: HR-ESIMS data for pachymic acid (**12**)

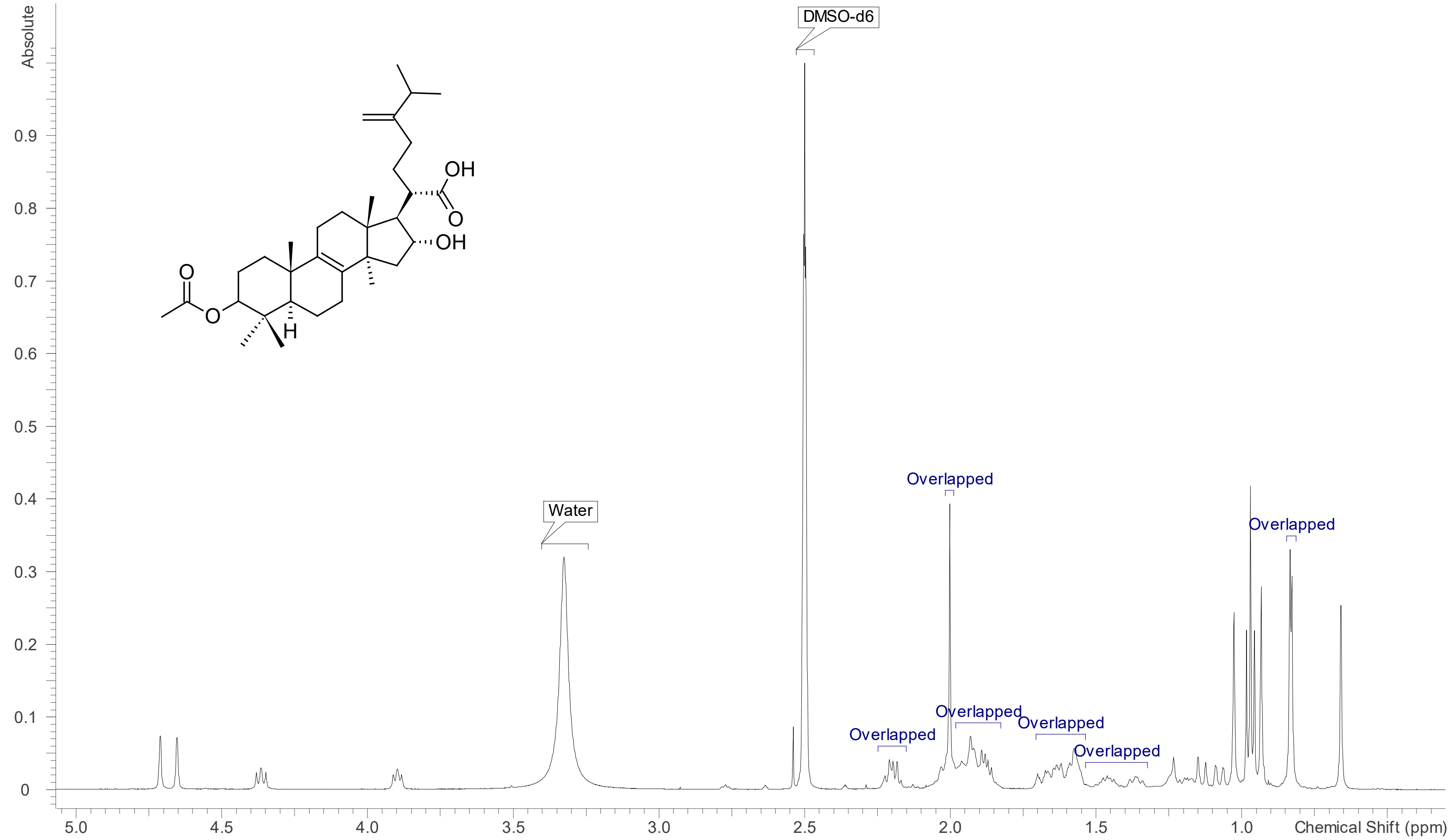

Figure 28: 1H NMR spectrum (DMSO- d_6, 700 MHz) of pachymic acid (**12**).

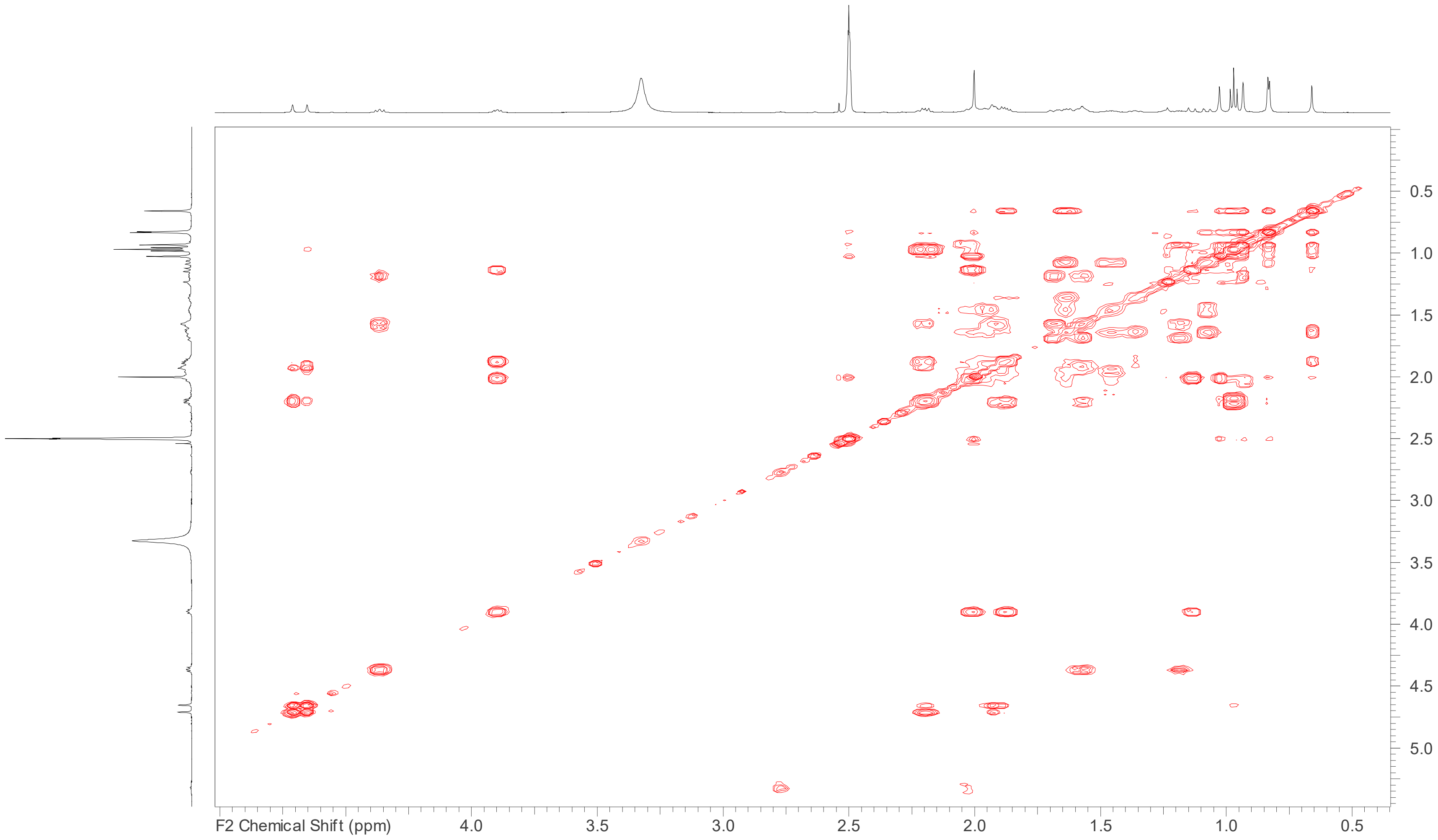

Figure S29: COSY spectrum (DMSO-d_6, 700 MHz) of pachymic acid (**12**).

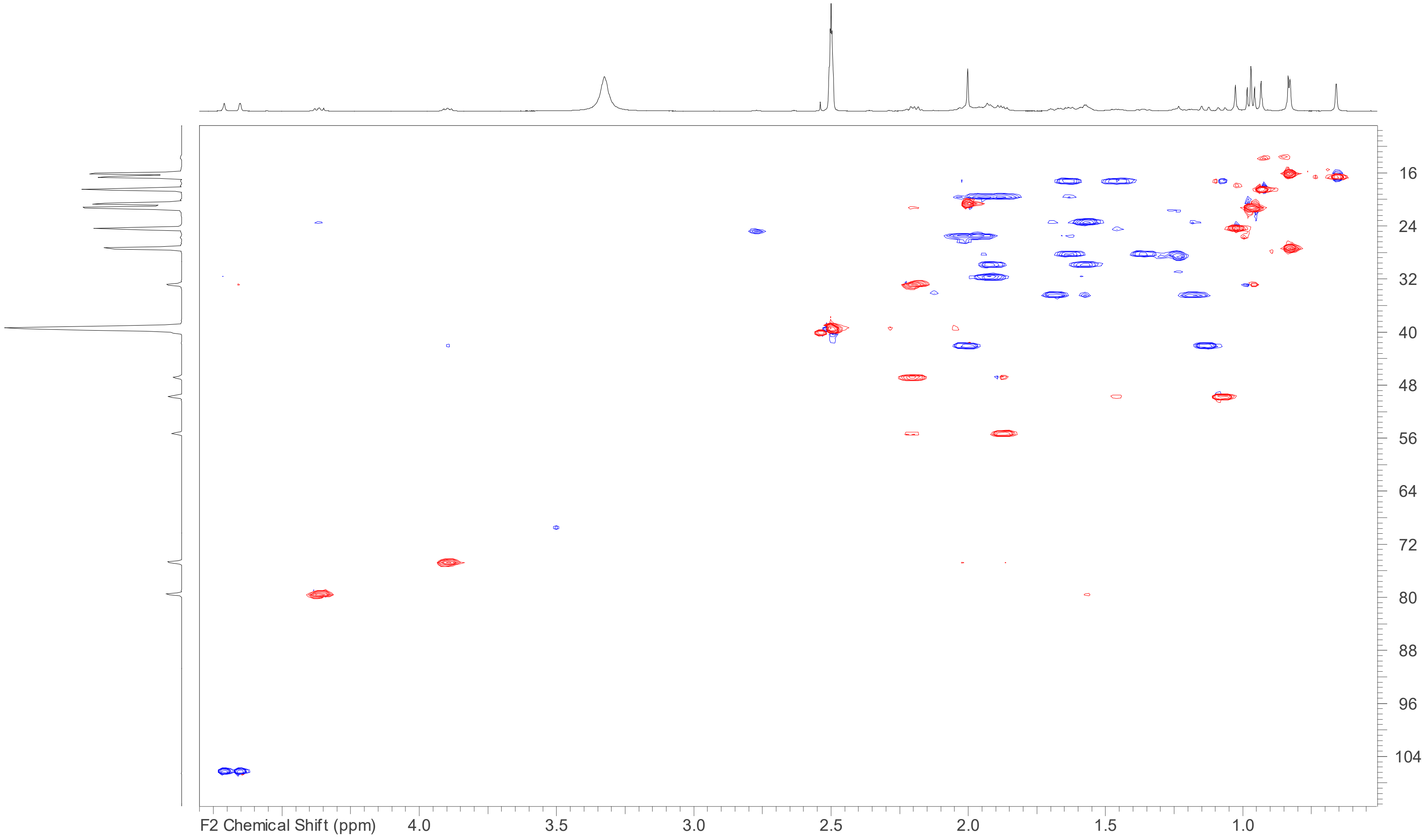

Figure S30: HSQC spectrum (DMSO-d_6, 700 MHz) of pachymic acid (**12**).

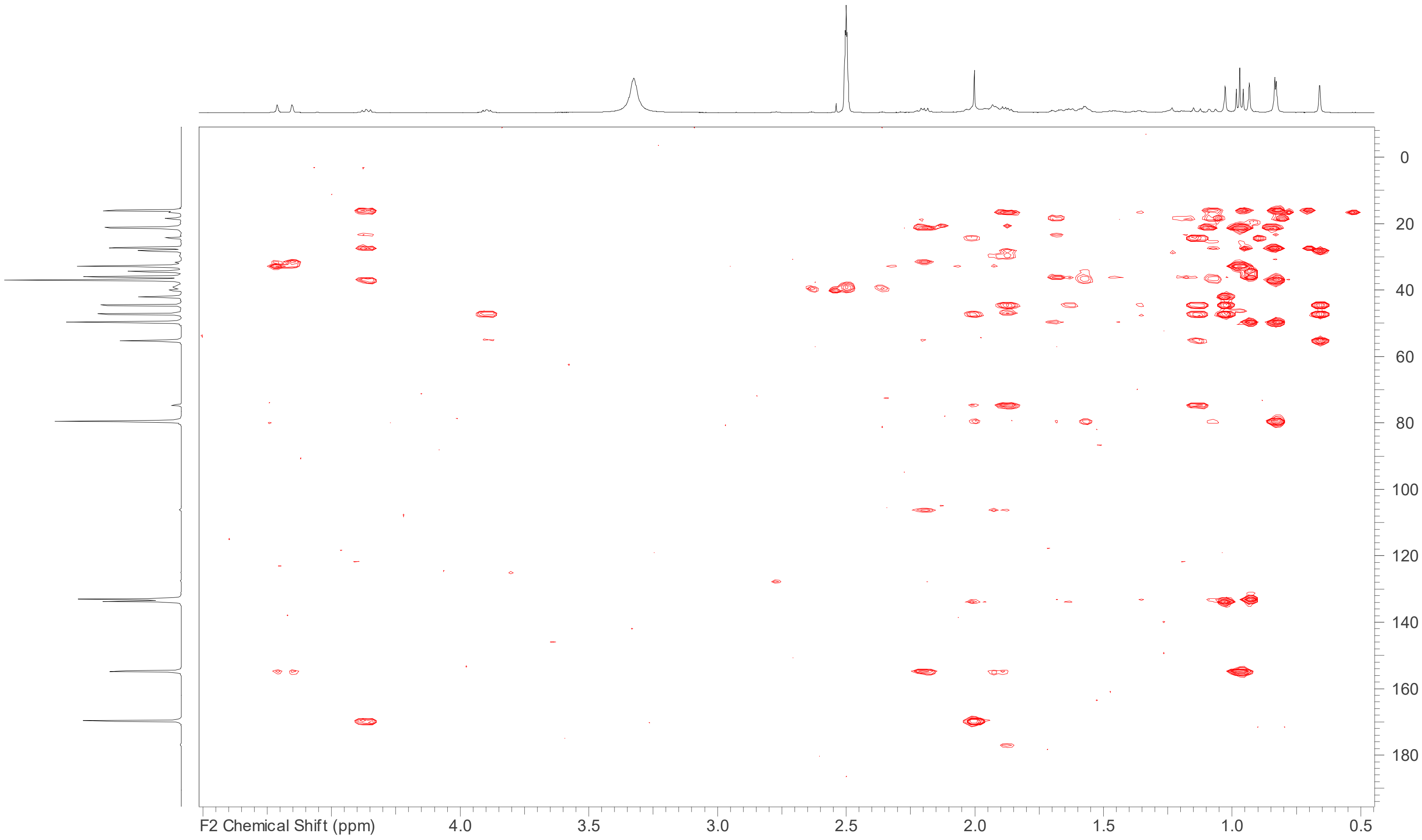

Figure S31: HMBC spectrum (DMSO-d_6, 700 MHz) of pachymic acid (**12**).

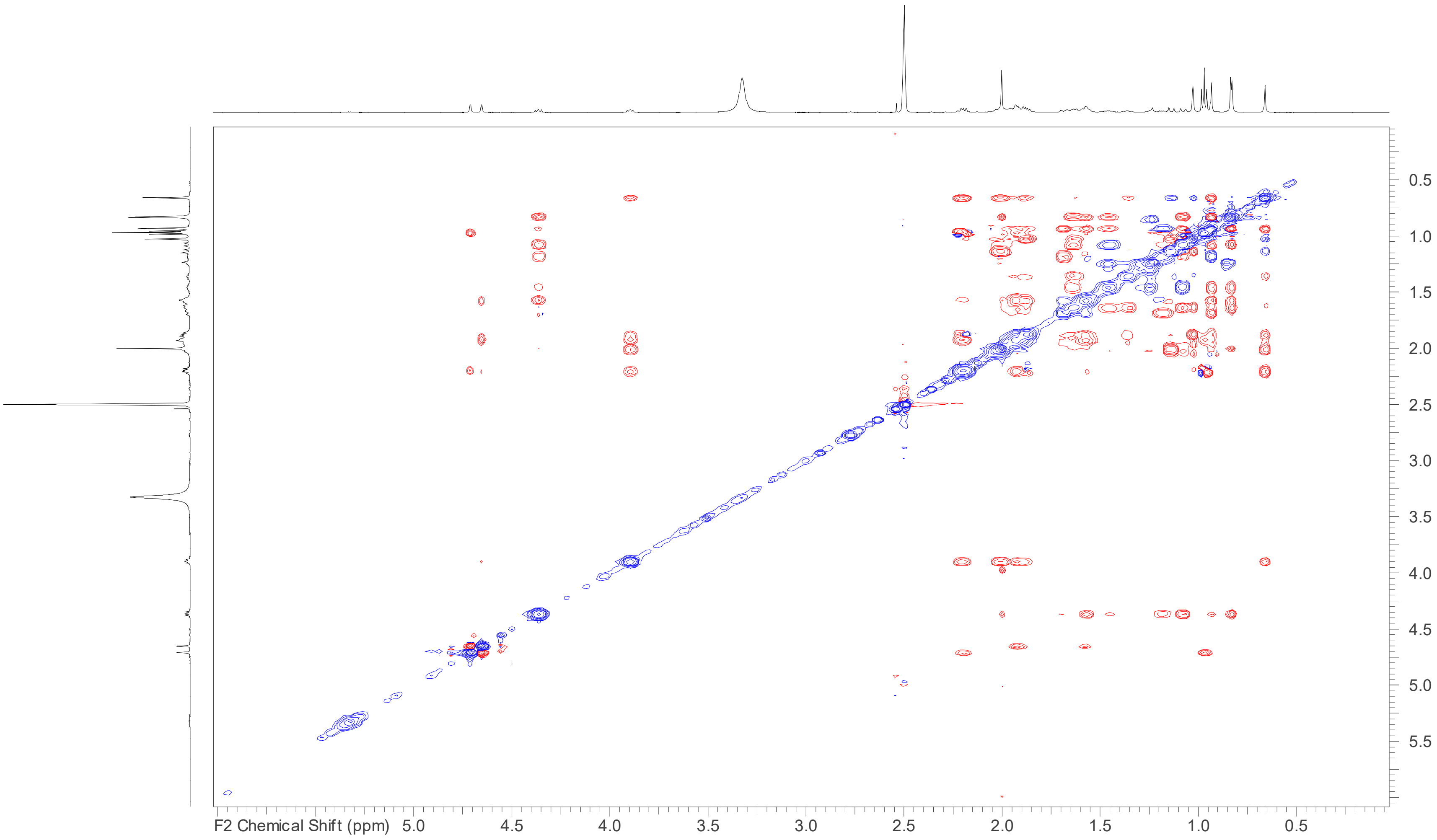

Figure S32: ROESY spectrum (DMSO- d_6, 700 MHz) of pachymic acid (**12**).

ITS and LSU sequences of *Antrodia* sp.

> LSU

GGATTCCCCTAGTAACTGCGAGTGAAGCGGGAAGAGCTCAAATTTAAAATCTGGCGGTCTCTGGCCGTCCGAGTTGTATTCTGGAGAAGTGTTTTCCGTGCTGGA
CCGTGTACAAGTCTCTTGGAACAGAGCGTCATAGAGGGTGAGAATCCCGTCTTTGACACGGACTGCCAGTGCTTTGTGATGCGCTCTCAAAGAGTCGAGTTGTTT
GGGAATGCAGCTCAAAATGGGTGGTAAATTCCATCTAAAGCTAAATATTGGCGAGAGACCGATAGCGAACAAGTACCGTGAGGGAAAGATGAAAAGCACTTTG
GAAAGAGAGTTAAACAGTACGTGAAATTGCTGAAAGGGAAACGCTTGAAGTCAGTCGCGTTGATCAGGACTCAGCCTTGCTTTTGCTTGGTGCATTTTCTGGTTG
ACGGGCCAGCATCGATTTTGACCGTCGGAAAAGGGCTGAGGGAATGTGGCACCTTCGGGTGTGTTATAGCCTTCAGTCACATACGGCGATWGGGATCGAGGAC
CGCAGCACGCCTTTATGGCCGGGGTTCGCCCACGTTCGTGCTTAGGATGCTGGCGTAATGGCTTTAAACGACCCGTCTTGAAACACGGACCAAGGAGTCTAACAT
GCCTGCGAGTGTTTGGGTGGAAAACCCGAGCGCGCAATGAAAGTGAAAAGTTGAGATCCCTGTCATGGGGAGCATCGACGCCCGGGCCTGAACTTCGGTGATG
GTTCTGCGGTGGAGCATGTATGTTGGGACCCGAAAGATGGTGAACTATGCCTGAATAGGGTGAAGCCAGAGGAAACTCTGGTGGAGGCTCGTAGCGATTCTGA
CGTGCAAATCGATCGTCAAATTTGGGTATAGGGGCGAAAGACTAATCGAACCATCTAGTAGCTGGTTCCTGCCGAAGTTTCCCTCAGGATAGCAGAAACTCGTAT
CAGATTTATGTGGTAAAGCGAATGATTAGAGGCCTTGGGGTTGAAACAACCTTAACCTATTCTCAAACTTTAAATATGTAAGAACAACCCGTCACTTGATTGG

> ITS

AGTTCAGCGGGTAATCCTACCTGATCTGAGGTCAAAGGTCAAGATAAATTGTCCTTTAGCAGGACGATTAAGAAGCTGACACCCATACAACATGCTTCACAGAAC
AGTGTAAACAAATTATCACACTGAAGCTGATTCACAAAAGGTTTCAAGCTAATGCATTCAAGAGGAGCTGAACACAGTAGTATCCAGCACACTCCAAATCCAAGC
TCCATTCACAGAAATGAATAGAGTTGAGAATTCCATGACACTCAAACAGGCATGCTCCTCGGAATACCAAGGAGCGCAAGGTGCGTTCAAAGATTCGATGATTCA
CTGAATTCTGCAATTCACATTACTTATCGCATTTCGCTGCGTTCTTCATCGATGCGAGAGCCAAGAGATCCGTTGCTGAAAGTTGTATATAGATGCGTTACACGCA
ATAGACATTCTTTAAACTGAGTTGTGTGTGGGTAAAAACATAGGAAAGACCACAGAGCAAAGTCAATGAAGACTTCACTCCAAGAGCCTAATCTACAGTGTGTGC
ACAGGTGTGAGAGATGGATAATGATCAGGGTGTGCACAATGCCGCAGCCAGCAACAACCCCTTTCAAGATTCATTAATGATCCTTCCGCAGGTTCACCTACGGAA
ACC
